# Data augmentation and multimodal learning for predicting drug response in patient-derived xenografts from gene expressions and histology images

**DOI:** 10.3389/fmed.2023.1058919

**Published:** 2023-03-07

**Authors:** Alexander Partin, Thomas Brettin, Yitan Zhu, James M. Dolezal, Sara Kochanny, Alexander T. Pearson, Maulik Shukla, Yvonne A. Evrard, James H. Doroshow, Rick L. Stevens

**Affiliations:** ^1^Division of Data Science and Learning, Argonne National Laboratory, Lemont, IL, United States; ^2^Section of Hematology/Oncology, Department of Medicine, University of Chicago Medical Center, Chicago, IL, United States; ^3^Frederick National Laboratory for Cancer Research, Leidos Biomedical Research, Inc., Frederick, MD, United States; ^4^Division of Cancer Therapeutics and Diagnosis, National Cancer Institute, Bethesda, MD, United States; ^5^Department of Computer Science, The University of Chicago, Chicago, IL, United States

**Keywords:** drug response prediction, histology whole-slide images, gene expression, multimodal deep learning, preclinical drug studies, data augmentation, patient-derived xenograft (PDX)

## Abstract

Patient-derived xenografts (PDXs) are an appealing platform for preclinical drug studies. A primary challenge in modeling drug response prediction (DRP) with PDXs and neural networks (NNs) is the limited number of drug response samples. We investigate multimodal neural network (MM-Net) and data augmentation for DRP in PDXs. The MM-Net learns to predict response using drug descriptors, gene expressions (GE), and histology whole-slide images (WSIs). We explore whether combining WSIs with GE improves predictions as compared with models that use GE alone. We propose two data augmentation methods which allow us training multimodal and unimodal NNs without changing architectures with a single larger dataset: 1) combine single-drug and drug-pair treatments by homogenizing drug representations, and 2) augment drug-pairs which doubles the sample size of all drug-pair samples. Unimodal NNs which use GE are compared to assess the contribution of data augmentation. The NN that uses the original and the augmented drug-pair treatments as well as single-drug treatments outperforms NNs that ignore either the augmented drug-pairs or the single-drug treatments. In assessing the multimodal learning based on the MCC metric, MM-Net outperforms all the baselines. Our results show that data augmentation and integration of histology images with GE can improve prediction performance of drug response in PDXs.

## 1. Introduction

With recent advancements in applications of artificial intelligence in medicine and biology, predictive modeling has gradually become one of the primary directions in cancer research for analytically predicting the response of tumors to anticancer treatments (1, 2). In particular, conventional machine learning (ML) and deep learning (DL) methods have been widely investigated for building computational drug response prediction models for cancer cell lines with large datasets of omics profiles (3). The complex heterogeneities of cancer that occur within and between tumors present a major obstacle to successful discovery of robust biomarkers and therapies (4, 5). Patient-derived tumor xenografts (PDXs) are a contemporary biological model that is created by grafting cancerous tissue, obtained from human tumor specimens, into immunodeficient mice. The *in vivo* environment of PDXs helps preserve tumor heterogeneity as compared to *in vitro* cell lines, and therefore, is presumed to better mimic the response of human patients with certain cancer types. PDXs continue to gain reputation for studying cancer and investigating drug response in preclinical drug studies (6–8).

Predicting the response of tumors to drug treatments with accurate and robust computational models provides a modern approach for identifying top candidates for preclinical drug screening experiments or personalized cancer treatments. A variety of ML and DL approaches have been explored with high-throughput drug screens and cell lines (9, 10). Alternatively, our literature search retrieved only two publications that have used only PDX data to train prediction models for drug response (11, 12). Both studies used the Novartis PDX data (NIBR PDXE), which were generated using a 1 x 1 x 1 experimental design (13, 14), where each drug was tested against each patient PDX model using only one entumored mouse per model. Nguyen et al. (11) used an optimal model complexity (OMC) strategy with random forests to build drug response models for 26 treatment-cancer type combinations. They considered three genomic feature types in their analyses, including gene expressions (GE), copy-number alterations (CNAs), and single-nucleotide variants (SNVs). While considering a single feature type at a time, they used OMC to determine an optimal subset of features to obtain the best performing model for each treatment-cancer type pair. They showed that for the majority of cases, models developed with OMC outperform models that used all the available features. In another study, Kim et al. proposed PDXGEM, a pipeline that identifies biomarkers predictive of drug response in PDX and then uses the identified markers to train prediction models (12). To identify predictive genes based on GE and drug response, the pipeline utilizes a strategy similar to co-expression extrapolation (COXEN) (15, 16), and consequently selects the genes whose co-expression patterns are best preserved between PDXs and patient tumors. They trained prediction models using random forests for six treatment-tumor type combinations and then predicted response in patients.

A primary challenge in modeling drug response with PDXs is the limited availability of drug response data. The sample size of PDXs is usually orders of magnitude smaller than the analogous cell line datasets. It has been shown that increasing the amount of training samples improves generalization performance of supervised learning models in vision and text applications (17, 18), as well as drug response models in cell lines (19, 20). Collecting PDX response data, either through experiments or integration of multiple datasets, carries considerable technical and financial challenges. Alternatively, instead of directly increasing the sample size, the volume of data can be expanded by representing each sample with multiple feature types. Multimodal architectures that integrate genomic and histology images have been shown to improve prognosis prediction of patients with cancer as compared with unimodal architectures that learn only from a single data modality (i.e., feature type) (21–23). Another possible direction to address the limited sample size is data augmentation. Augmentation techniques have been extensively explored with image and text data, but not much with drug response. While augmenting images has become a common practice, tabular datasets such as omics profiles lack standardized augmentation methods.

In this study, we investigate two approaches for predicting drug response in PDX, including multimodal learning and data augmentation. We explore a multimodal neural network (MM-Net) that learns to predict drug response in PDXs using GE and histology whole-slide images (WSIs), two feature types representing cancer tissue, and molecular descriptors that represent drugs. The multimodal architecture is designed to take four feature sets as inputs: (1) GE, (2) histology images, and (3,4) molecular descriptors of a pair of drugs. We benchmark the prediction performance of MM-Net against three baselines: (1) NN trained with drug descriptors and GE, (2) NN trained with drug descriptors and WSIs, and (3) LightGBM model (24) trained with drug descriptors and GE. With multimodal learning, our goal is to explore whether the integration of histology images with GE improves the prediction performance as compared with models that use GE features alone. For data augmentation, we homogenize the drug representation of single-drug and drug-pair treatments in order to combine them into a single dataset. Moreover, we introduce an augmentation method that doubles the sample size of all drug-pair treatments. The proposed augmentations allow us to combine single-drug and drug-pair treatments to train MM-Net and the baselines without changing the architectures and the dataset. We explore the contribution of augmented data for improving the prediction of drug response.

This paper provides unique contributions compared with existing works that train drug response models with PDX data (11, 12). First, we build general drug response models for PDXs across multiple cancer types and drug treatments. Alternatively, prediction models in Kim et al. (12) and Nguyen et al. (11) are built for specific combinations of cancer type and drug treatment. Thus, our study targets a more challenging task. Second, we utilize PDX histology images with multimodal architecture which has not yet been studied for drug response prediction in PDX. Our study presents a framework for integrating image data with genomic measurements and drug chemical structure for predicting treatment effect. Third, we combine multiple treatments into a single dataset by homogenizing single-drug and drug-pair treatments and utilize drug features for training models. This provides an advantage over existing works which built prediction models for unique combinations of drugs and cancer types, and therefore, disregard drug features when model training. Furthermore, we propose an augmentation method for drug-pairs that doubles the sample size of the drug-pair treatments in the dataset. Fourth, existing studies built prediction models using the PDXE drug screening data which were generated using a 1 x 1 x 1 experimental design, i.e., one mouse per model per treatment. In contrast, we utilized the PDMR dataset where treatment response is measured by comparing a group of treated mice to a group of untreated mice. The group approach allows assessing the variability of response across mice and might be considered as more reliable in capturing tumor heterogeneity.

## 2. Materials and methods

### 2.1. Data

#### 2.1.1. Experimental design of drug efficacy in PDX

**Figure 1:**
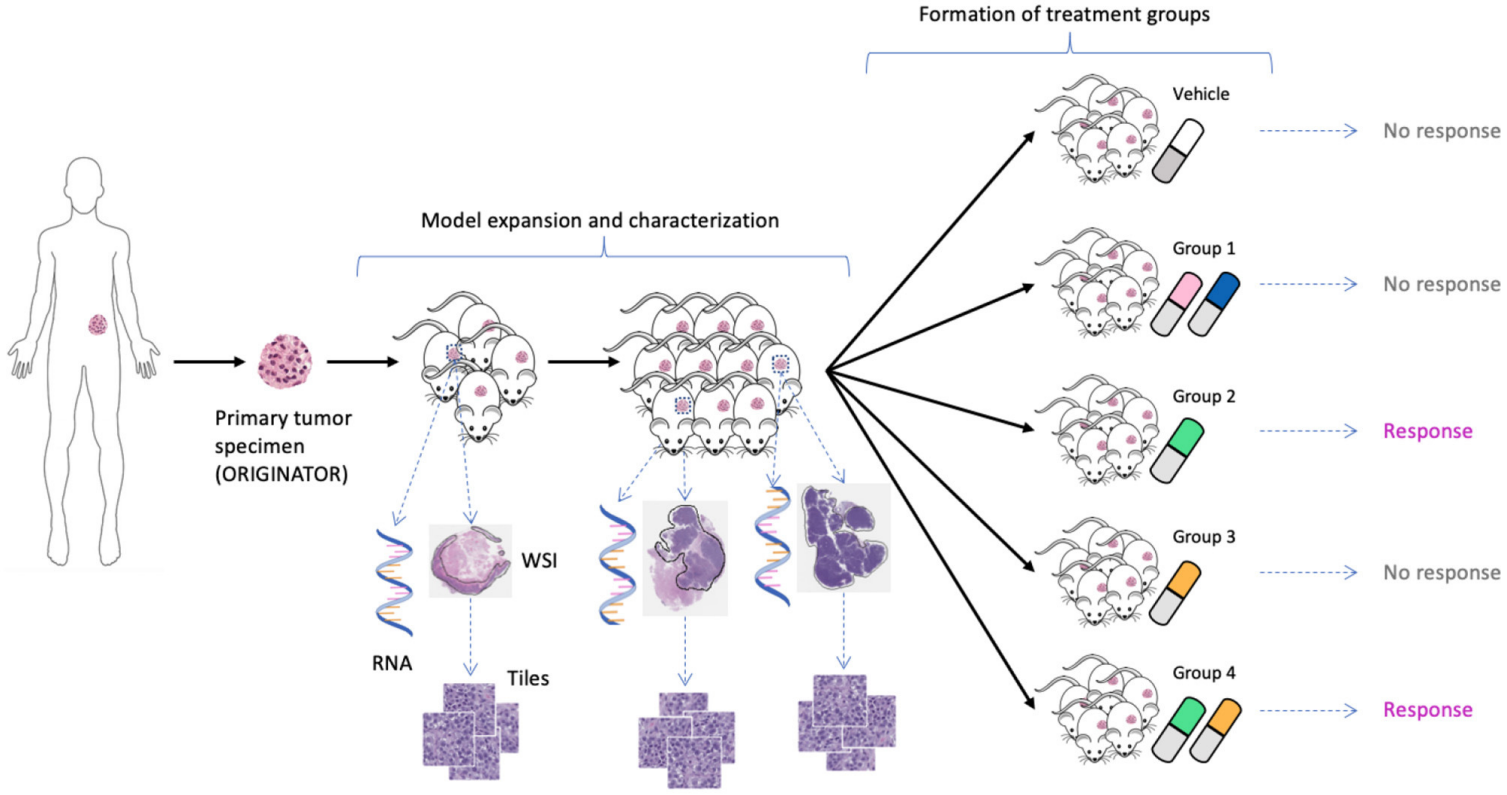
Expansion of tumor tissue from the source specimen (ORIGINATOR) to mice across multiple passages. Mice originated from the same specimen are divided into a control group and multiple treatment groups. Tumors from certain mice were histologically and molecularly profiled, resulting in whole-slide images and omics profiles.

We used unpublished PDX drug response data from the NCI Patient-Derived Models Repository (PDMR; http://pdmr.cancer.gov). The NCI PDMR performs histopathology assessment, whole-exome sequencing and RNA-Seq analysis of a subset of tumors from each PDX model to establish baseline histology and omic characterization for each model. To date, over six hundred unique PDX models have been characterized and data made available through the public website. Baseline pathology and omic characterization from 487 models were used for this analysis. The efficacy of drug treatments in PDMR is measured through controlled group experiments. Figure 1 illustrates the process of obtaining primary tumor specimen from a patient, engrafting tumor tissue into PDX models, performing baseline characterization, expanding tumor tissue over multiple passages within a lineage, and then using the expanded tumors in drug treatment experiments. A total of 96 PDX models from 89 unique patients were used for the experiments. The tumors are grown subcutaneously in NOD.Cg-Prkdcscid Il2rgtm1Wjl/SzJ (NSG) host mice and staged to an approximate tumor weight of 200 mm^3^ for the drug studies. The control group is treated with a vehicle only (i.e., a solution that delivers drugs to the treated animals). The preclinical dataset includes twelve single-drug and 36 drug-pair treatment arms (the drugs are still in preclinical and/or clinical investigations and their names and properties are expected to be released in the future). Median tumor volume over time for each vehicle or treatment group is used for response assessment. The GE profiles and WSIs were aggregated and preprocessed for the downstream ML and DL analysis.

#### 2.1.2. Drug response in PDX

**Figure 2:**
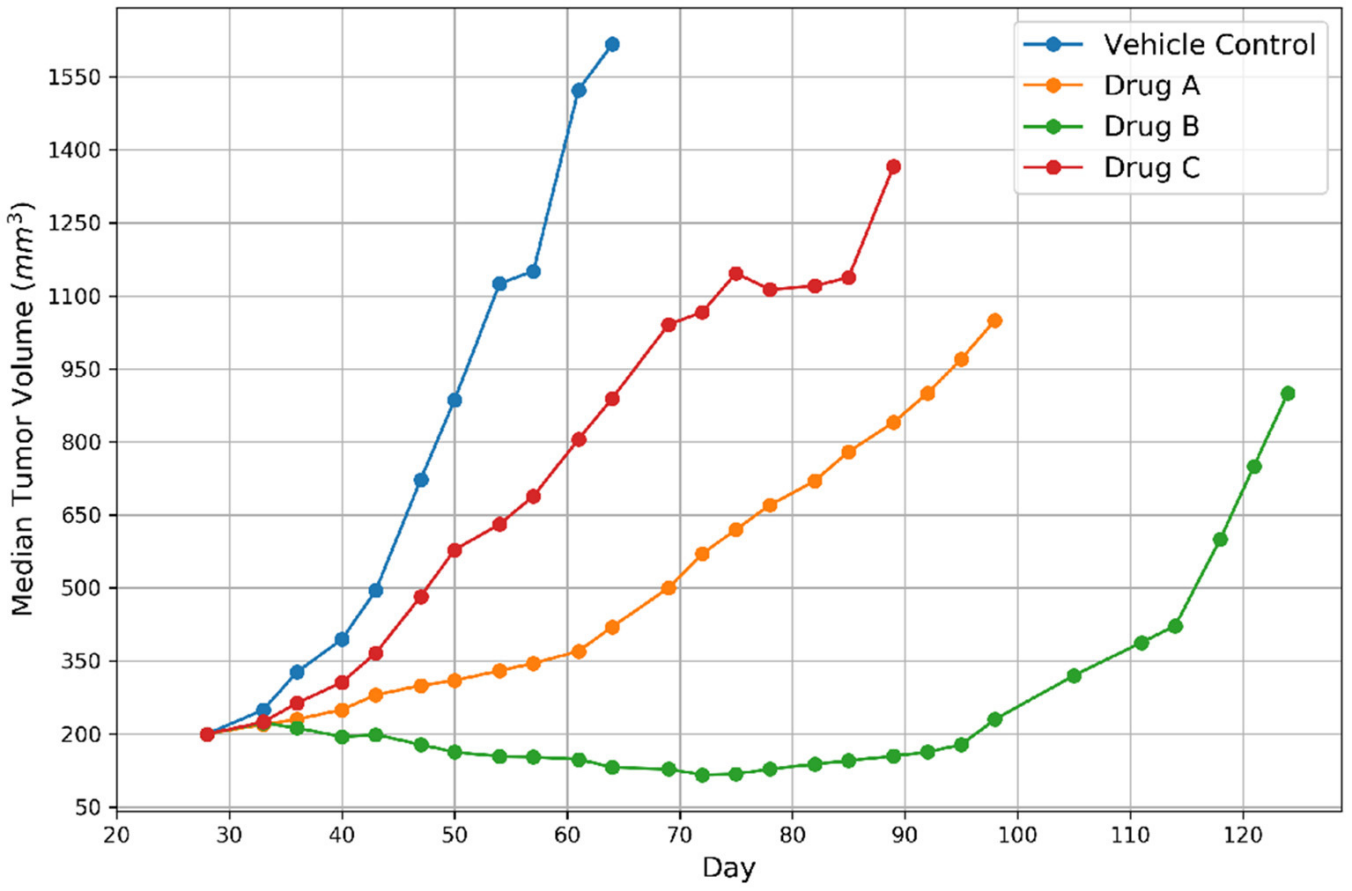
Representative tumor growth curves for vehicle control and three drugs. The label of response was assigned to Drug B because the tumor achieved regression and non-response was assigned to the remaining drugs.

The growth of tumor volume over time represents the response of PDX tumors to drug treatments, as shown in Figure 2. There are several methods available in the literature for encoding drug response in PDXs which include both continuous and categorical types, but no consensus currently exists regarding which type or actual representation is better (6, 25). Continuous metrics include percent change in tumor volume, area under the tumor growth curve, best tumor response, best average response, and more. In this study we chose to use the binary representation of response which aligns with other drug response prediction studies that are mentioned earlier (11, 12), and the RECIST criteria, an existing standard for encoding response in patients (26). The group approach intends to capture the variability of PDX drug response across mice of the same lineage (25). Median tumor volume per treatment group is assessed relative to the control group to create a binary variable representing response. Specifically, for each drug treatment experiment, a single experienced preclinical study analyst assessed the curves of median tumor volume over time for each treatment arm, and assigned label “1” for response (regression of at least 30% from staging for more than one consecutive time point at any point during the study) and label “0” for non-response. In essence, a modified RECIST score for regression vs. no regression was used to label the response. Thus, a single best response value was assigned for each treatment arm.

#### 2.1.3. Data generation

Three feature types were used for model training, including drug descriptors, GE, and histology image tiles.

**Gene expressions**. Gene expressions have been considered to provide more predictive power than other omics data types for drug response prediction (DRP) (27), and therefore, are often used to represent cancer in DRP models, either standalone or in a combination with other multiomics (28). However, the high dimensionality of gene expressions and the relatively small sample size can lead to overfitting (2). To address this issue, several gene selection methods have been utilized, including filtering genes based on variability across samples (29–31) or using gene subsets such as LINCS (10, 32–36) or COSMIC (35, 37–40) that are known to be associated with cancer and/or treatment response. We are not aware of any systematic analysis that studied which filtering method better addresses overfitting and improves prediction generalization. In this study, we filtered the RNA-seq data by selecting 942 landmark genes discovered by the Library of Integrated Network-Based Cellular Signatures (LINCS) project. The LINCS genes have been shown to comprehensively characterize and infer the gene expression variation of more than 80% of the whole transcriptome (32). We used TPM (transcripts per kilobase million) expression values of these genes, which were transformed by *log2x+1*, where *x* is the TPM value of a gene. The *log* transformed TPM values of each gene were then standardized to have a zero mean and a unit standard deviation across all gene expression profiles.

**Drug descriptors**. We used the Dragon software package (version 7.0) to calculate numerical descriptors of drug molecular structure. The software calculates various types of molecular descriptors, such as atom types, estimations of molecular properties, topological and geometrical descriptors, functional groups and fragment counts, and drug-like indices. A total of 1,993 descriptors were used for the analysis after removing descriptors with missing values. We standardized the descriptor values across drugs to have a zero mean and a unit standard deviation.

**Histology images**. During PDX model expansion, entumored mice were sacrificed between 1,000 and 2,000 mm^3^ for collection of tumors for representative model characterization including histopathological examination. Hematoxylin and Eosin (H&E) stained pathology slides were digitized into WSIs using an Aperio AT2 digital whole slide scanner (Leica Biosystems) at 20x magnification. A board-certified pathologist from Frederick National Laboratory of Cancer Research reviewed the slides to ensure the PDX models were consistent with the original patient diagnosis. Tumor regions of interest (ROIs) were annotated within the image slides using QuPath (41) by a single University of Chicago pathologist.

**Figure 3:**
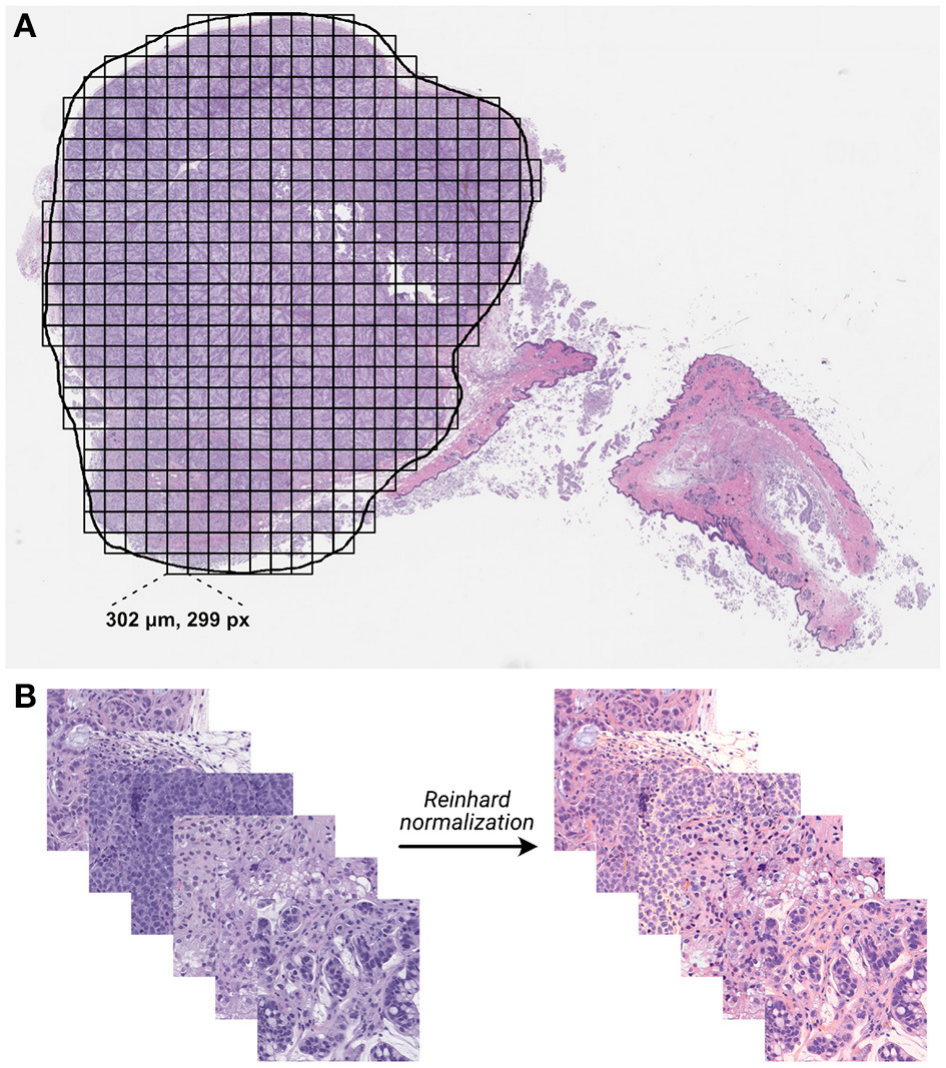
Whole-slide histology image processing. **(A)** Whole-slide images were annotated with region of interest (ROI) outlines, and image tiles were extracted from within ROIs in a grid-wise fashion. **(B)** Extracted non-background tiles underwent digital stain normalization using the Reinhard method (44).

Whole slide images were processed into individual tiles using the Slideflow software package (42, 43), as shown in Figure 3. Image tiles were extracted from within annotated ROIs in a grid pattern at 302 μm by 302 μm with no overlap, then downsampled to 299 pixels by 299 pixels, resulting in an effective optical magnification of 10x. Background tiles were removed with grayscale filtering, where each tile is converted to the HSV color space and removed if more than 60% of its pixels have a hue value of less than 0.05. Image tiles then underwent digital stain normalization using the Reinhard method (44) and were subsequently standardized to give each image a mean of zero with a variance of one.

#### 2.1.4. Constructing PDX drug response dataset

**Figure 4:**
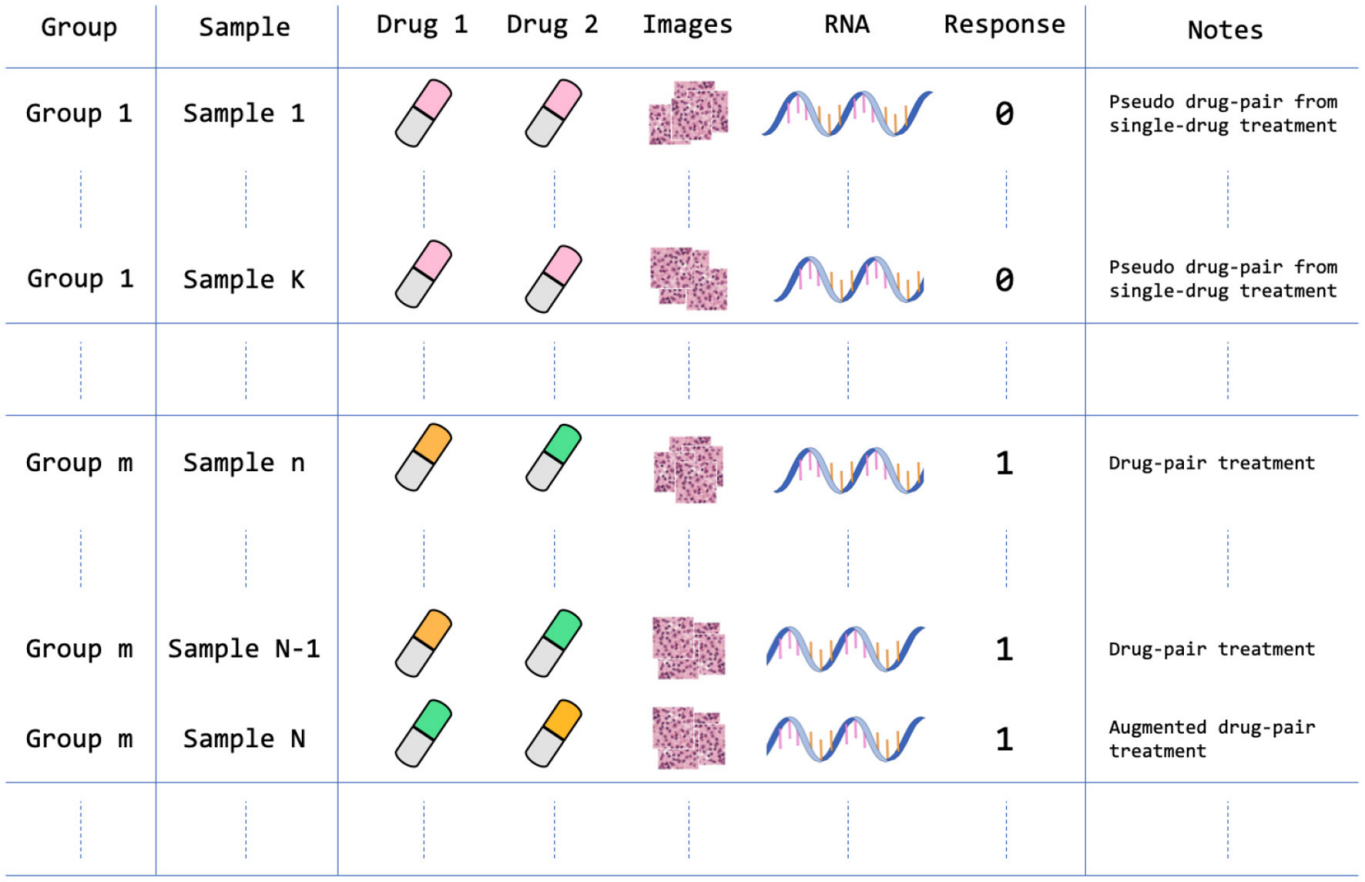
Data arrangement of the PDX drug response dataset. The dataset contains 959 treatment groups after homogenizing and augmenting the drug experiments as described in Section 2.1.4. For example, Sample 1 is a single-drug treatment that is structured as a pseudo drug-pair treatment where Drug 1 and Drug 2 features are the same feature vectors; Sample N is an augmented version of Sample N-1, in which the positions of drug feature vectors are switched. Note that each sample contains multiple histology image tiles that were extracted from a large WSI.

In constructing the drug response dataset, we populated samples from each group experiment with the corresponding response label. Each sample that was molecularly and histologically profiled consists of three feature types and a binary response value. The feature types include drug descriptors, GE, and histology tiles, as illustrated in Figure 4. Table 1 lists the summary statistics of the dataset.

**Table 1:** Summary of the PDX drug response dataset used for building prediction models.

Patients Primary tumor specimens	89 96
Single-drug treatments Drug-pair treatments (excludes augmented samples) Treatment groups	12 36 959
Gene expression profiles Histology whole-slide images (WSIs) Histology image tiles (extracted from WSIs)	487 487 177,468
Single-drug response values Drug-pair response values (includes augmented values) Drug response values	2,556 4,4406 6,962

The PDMR preclinical dataset contains experiments of single-drug treatments and drug pairs. In order to include both single-drug and drug-pair treatments in the dataset, and ensure consistent dimensionality of drug features, we homogenized single-drug treatments by duplicating drug descriptors to form a pseudo drug-pair that includes two identical drug feature vectors. In this case, the samples of single-drug and drug-pair treatments will follow the same input dimensionality for all ML models. Moreover, because switching the position of drug features in drug-pair treatments should not change the drug response, we augmented all drug-pair samples by switching the position of the two drugs while keeping the drug response value unchanged. Such data augmentation doubles the number of drug-pair samples in the dataset.

Following the integration of group samples into the dataset and the augmentation of drug-pair treatments, the drug response dataset contains 6,962 samples. The total number of treatment groups in the dataset is 959 with 917 non-response and 42 response groups. The dataset contains three feature types (modalities): two vectors of drug descriptors (a vector for each drug), GE profile, and histology tiles. Each sample consists of a unique combination of drug descriptors and GE profiles. However, each such sample contains multiple image tiles from a corresponding histology slide. Concretely, each sample consists of a GE profile, vector of descriptors for drug 1 and drug 2, and multiple image tiles as shown in Figure 4. We store the data in TFRecords (TensorFlow file format) which enables efficient data prefetching and loading, and therefore, considerably decreases the training and inference time.

#### 2.1.5. Data splits

Data leakage can lead to overly optimistic predictions (45). Two primary characteristics of our dataset may lead to leakage if random splitting is used to generate training, validation, and test sets. First, a drug response label is assigned to all the samples in the entire treatment group. To prevent leakage, we make sure that samples from the same treatment group always appear together in one of the training, validation, or test sets. Second, the augmented drug-pair samples represent, in reality, the same experiment, and therefore, are also placed together when generating the splits. With this strategy, we generated 100 data splits for the analysis (10-fold cross-validation repeated ten times with different random seeds), where the tissue features (GE profiles and WSIs) of the same treatment group are kept together and not shared across training, validation, and test sets of each data split.

### 2.2. Prediction models

**Figure 5:**
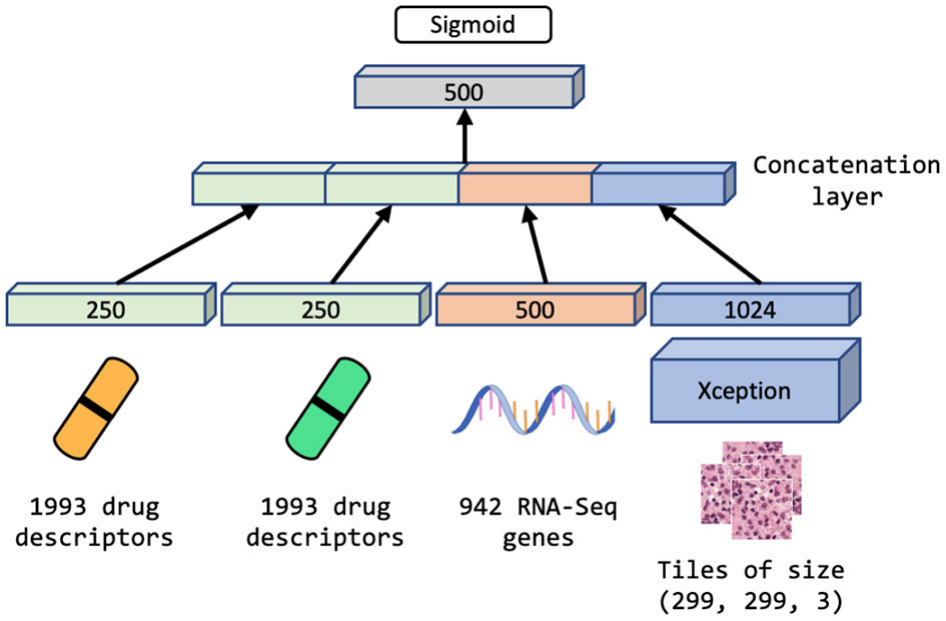
Multimodal neural network (MM-Net) learns from drug descriptors, gene expressions, and tiles generated from whole-slide images, to predict drug response in PDXs.

We explore the performance of MM-Net, shown in Figure 5, in predicting the drug response in PDXs. The model takes preprocessed feature sets as inputs, including drug descriptors, GE, and histology tiles, and passes them through subnetworks of layers. The encoded features from the subnetworks are merged *via* a concatenation layer and propagated to the output for predicting a binary drug response.

Since the dataset is highly redundant in terms of GE and drug features as shown in Figure 4 and Table 1 (there are 48 unique drug treatments and 487 unique expression sets), we use a single layer of trainable weights to encode these features with the goal to mitigate overfitting. The image tiles are passed through a subnetwork of convolutional layers of the Xception model (46) with weights pre-trained on ImageNet (47). The output from the convolutional neural network (CNN) is passed through a series of dense layers before being concatenated with the encoded GE and drug descriptor representations.

We compare the performance of MM-Net with three unimodal baselines that use either GE or WSI as tumor features: (1) UME-Net, NN that uses GE, (2) UMH-Net, NN that uses histology tiles, and (3) LGBM, LightGBM that uses GE. Note that all models use drug descriptors.

### 2.3. Training and evaluation

We used a randomized search to obtain a set of hyperparameters (HPs) for UME-Net, including optimizer, learning rate, and layer dimensions that encode GE and drug descriptors. The values of these HPs were also used for MM-Net. A few remaining HPs that are unique to MM-Net were determined in a separate search using the MM-Net architecture. Another randomized search was performed to obtain the HPs for LGBM such as the number of leaves in the decision tree and the number of trees.

To mitigate overfitting, we used the early stopping mechanism in TensorFlow and LightBGM where model trainings terminate automatically if the predictions on a validation set have not been improved for a predefined number of training iterations. The early stopping parameter was set to 10 epochs for all NNs and 100 boosting rounds for the LGBM. All NNs were trained for 400 training epochs which triggered early stopping and ensured model convergence. To further address overfitting, we applied standard image augmentation methods such as rotation and horizontal flipping to histology tiles during the training of MM-Net and UMH-Net.

Since the dataset is highly imbalanced in terms of drug response distribution, we used a weighted loss function that penalizes more heavily incorrect predictions of the response samples as opposed to the non-response samples. For training MM-Net, we used only 10% of the image tiles that were available in each WSI, because our preliminary experiments revealed that the prediction performance does not improve if additional tiles are used. The 10% of the tiles have been drawn at random from each WSI.

For model evaluation, we used three performance metrics for binary classification tasks, including Matthews correlation coefficient (MCC), area under the receiver operating characteristic curve (AUROC), and area under the precision-recall curve (AUPRC). We calculated the metrics based on a test set of each one of the 100 splits. To compute each metric for a given test set, we aggregated all the sample predictions in the test set. In the case of baseline models where each tumor sample is represented by a GE vector, the prediction model generates in a single probability value for each sample. However, in the case of MM-Net where each tumor sample is represented by multiple image tiles in addition to the GE profile, the prediction model generates a single probability value for each image tile which results in multiple predicted probability values for a single sample. To conform with the output of the baseline models, the tile predictions from MM-Net were aggregated *via* mean to provide a single probability value for each sample. Note that while only 10% of the available tiles were used for training MM-Net, all tiles in the test set were used to compute predictions and subsequently obtain the performance metrics.

## 3. Results

**Figure 6:**
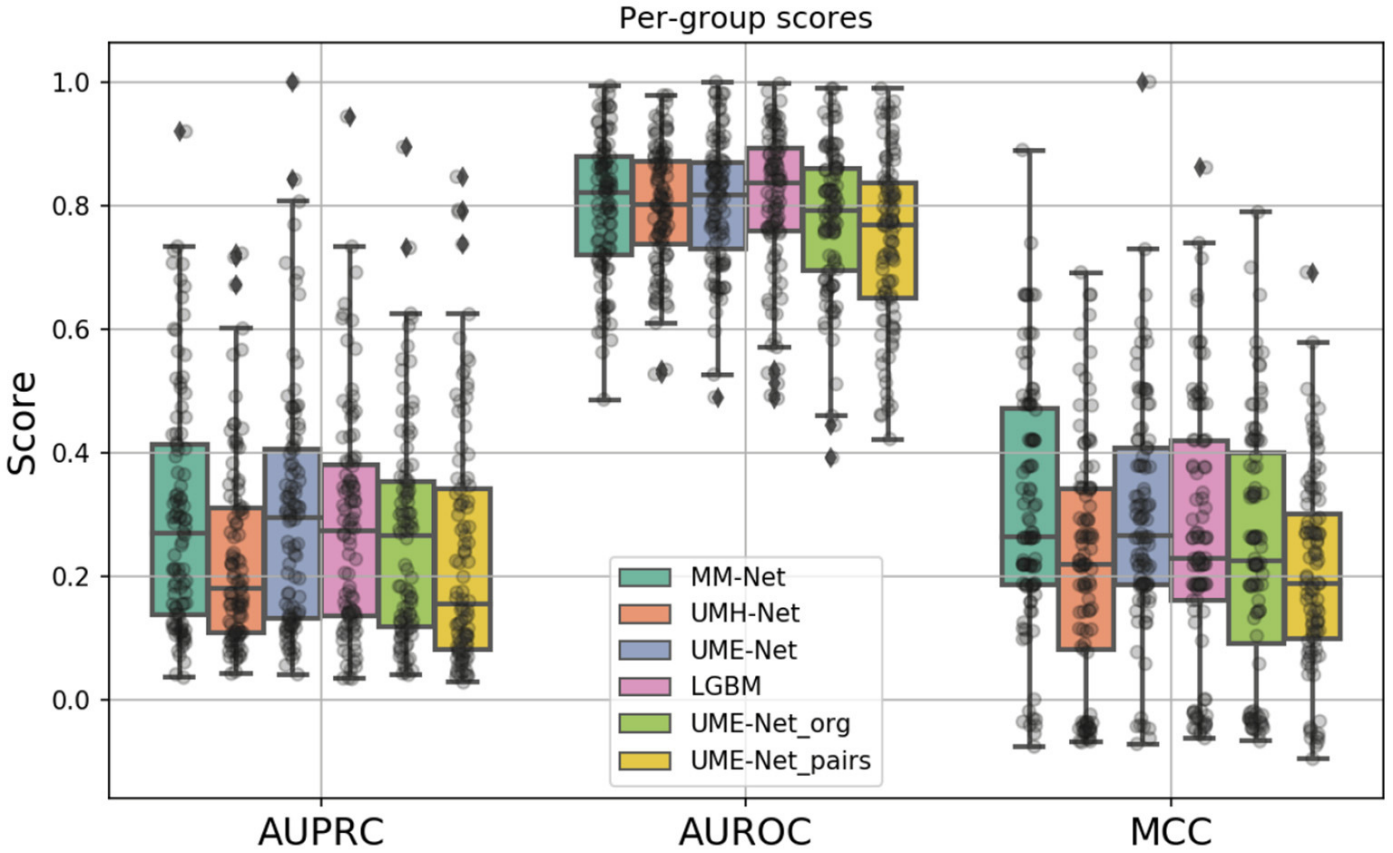
Boxplots showing the distribution of scores for the investigated drug response prediction models. The differences between the models are summarized in Table 2.

A total of six prediction models were analyzed, as summarized in Table 2. The models differ in terms of the feature sets and the samples that were used for training and validation (binary columns in Table 2). All models were evaluated across the same 100 data splits. Figure 6 shows the performance metrics, including MCC, AUPRC, and AUROC where each data point is a metric value calculated for a given split. The average score of each model was aggregated *via* mean across the splits for each metric (listed in Table 2).

**Table 2:** Performance metrics including MCC, AUPRC, and AUROC are listed for drug response prediction models (UME-Net, UME-Net_*org*_, UME-Net_*pairs*_, UMH-Net, MM-Net, and LGBM).

**Model**	**WSI**	**GE**	**Single-drug**	**Drug-pair**	**Augmented drug-pairs**	**MCC**	**AUPRC**	**AUROC**
UME-Net	–	v	v	v	v	0.2958	**0.2996**	0.8047
UME-Net_*org*_	–	v	v	v	–	0.2391	0.2610	0.7766
UME-Net_*pairs*_	–	v	–	v	v	0.2039	0.2355	0.7423
UMH-Net	v	–	v	v	v	0.2124	0.2303	0.7977
MM-Net	v	v	v	v	v	**0.3102**	0.2974	0.7978
LGBM	–	v	v	v	v	0.2594	0.2784	**0.8065**

As summarized in the binary columns (with v's), the differences between the models are in the feature sets (GE, WSI, or both) and the samples. To compute the average score for each metric and model, the predictions were aggregated *via* mean across the data splits.

Best scores per metric are shown in bold.

In constructing the drug response dataset, we used two approaches to increase the number of response values, as described in Section 2.1.4. We analyzed the effect of these two methods on the prediction performance by comparing three unimodal NNs that were trained with GE and drug descriptors on subsets of the dataset: (1) UME-Net, trained with the full dataset that includes the original and the augmented drug-pair treatments as well as single-drug treatments, (2) UME-Net_*pairs*_, trained with only drug-pair samples which include the original and the augmented samples, and (3) UME-Net_*org*_, trained with the original single-drug and drug-pair samples that exclude the augmented drug-pair samples. Figure 6 shows the prediction performance of using the different training subsets across the data splits where each data point is a metric value calculated for a given split. While the distribution of scores across the splits is quite substantial, removing either subset of samples (i.e., single-drug samples or augmented drug-pairs) results in a significant decline in prediction performance, as demonstrated by two statistical tests, including paired *t*-test and Wilcoxon signed-rank test (*p* < 0.05). In other words, augmentation methods lead to a significant improvement in performance of the NNs when trained with GE and descriptors. Hence, we used the full set of the available training samples for the analyses of multimodal learning.

The MM-Net architecture, shown in Figure 5, was compared against three baseline models, including UME-Net, UMH-Net, and LGBM. The performance metrics represent the ability of the models to generalize to a test set of unseen observations. Statistical tests (paired *t*-test and Wilcoxon signed-rank test) were performed to assess statistical difference between the models across the 100 data splits. Based on the aggregated MCC score, MM-Net statistically significantly outperforms all the baselines (*p* < 0.05 for both tests) except for UME-Net. When considering the AUPRC, MM-Net outperforms UMH-Net but there is no significant difference when comparing MM-Net with UME-Net or LGBM. No statistical significance was observed when comparing MM-Net with the other models. All the performance scores and statistical tests are provided in Supplementary Table S1.

**Figure 7:**
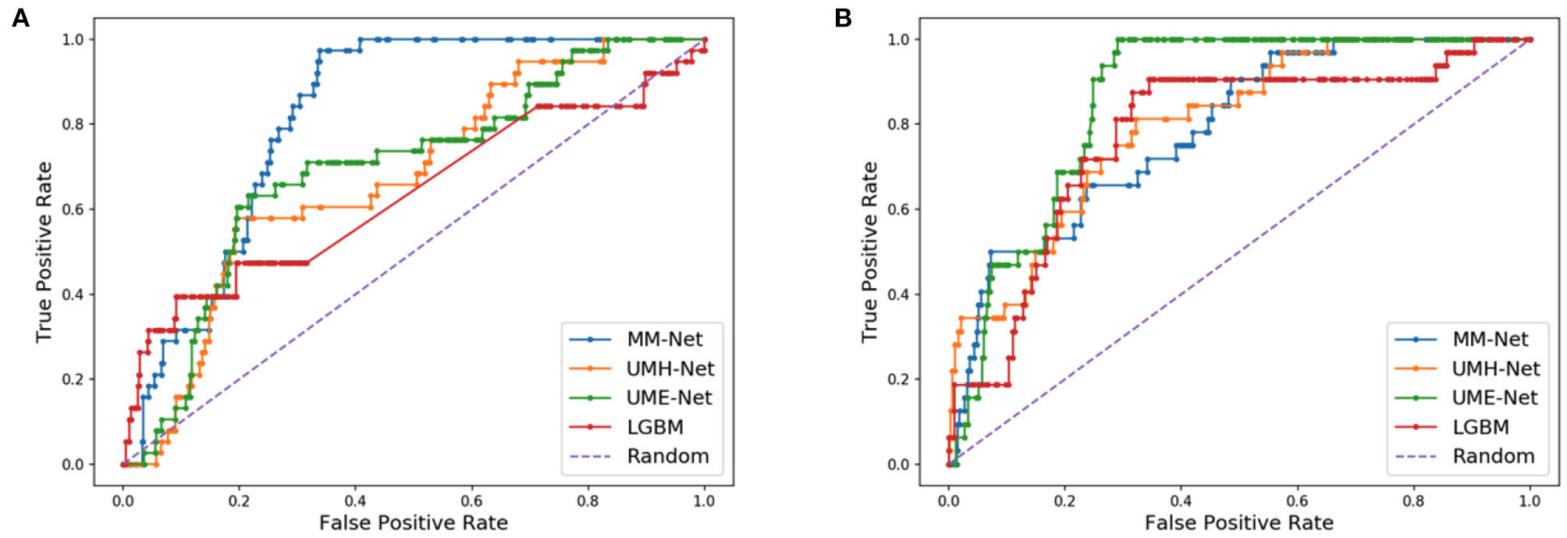
Receiver operating characteristic (ROC) curves for two different data splits. **(A)** MM-Net outperforms the baseline models. **(B)** MM-Net underperforms the baseline models.

As compared to DL models that are trained with cell line data, all models in Table 2 generally exhibit a relatively lower performance. For example, the average AUPRC is around 0.27 in Table 2 (all precision-recall curves are provided in the Supplementary material) but models trained on cell lines can exhibit AUPRC of 0.7 and above (48). Yet, we can observe a large spread of scores for all models and metrics (Figure 6). This indicates that for certain data splits, the models exhibit very high generalization performance, while for other splits, the models almost entirely fail to learn a meaningful mapping function for predicting drug response. In practical scenarios, where the goal is to design a highly generalizable model, a careful analysis should be conducted to determine the training and validation sets that adequately represent the test set. Subsequently, the threshold of the classifier can be determined depending on the error rate that the stakeholders can tolerate which will ultimately depend on the specific application that the model was designed for (e.g., precision oncology, drug development, etc.). In our analysis, however, the objective was to conduct large-scale trainings across multiple data splits and examine the overall capacity of MM-Net in predicting drug response across multiple cancer types and treatments. We observe that for certain dataset splits, MM-Net outperforms the baselines but for other splits it underperforms, as shown in Figure 7. An in-depth investigation is further required to understand in which cases MM-Net trained with WSI significantly improves prediction generalization.

## 4. Discussion

In this study, we investigated data augmentation methods and a multimodal architecture for predicting drug response in PDXs. We utilized the PDMR drug response dataset of single-drug and drug-pair treatments that were generated in controlled group experiments with PDX models of multiple cancer types. To assess the utility of the proposed methods, we conducted a large-scale analysis by training MM-Net and three baseline models over 100 data splits that contain GE profiles, histology image tiles, and molecular drug descriptors. We demonstrated that data augmentation methods lead to a significant improvement in drug response predictions across all performance metrics (MCC, AUPRC, and AUROC). Alternatively, the MM-Net model exhibits statistically significant improvement in prediction performance only when measured by the MCC.

The data splitting strategy and the choice of performance metrics play an important role in the downstream analysis when evaluating the utility of prediction models for practical applications. The dataset size and its diversity in terms of PDX models and treatments allowed us to generate multiple data splits while mitigating data leakage between training, validation, and test sets. Since each split comprises unique GE and histology images, we face a challenging prediction problem as opposed to a situation in which the samples are randomly split. Alternative splitting strategies may involve a careful choice of a single test set with the goal to reduce the distributional shift between training and test sets (49). Instead of carefully assembling the most representative test set, we chose to conduct a large-scale analysis to assess the empirical range of prediction performance with NNs and LGBM. The results show a large spread of scores across the splits, indicating that for certain data splits the models exhibit high prediction performance, while for other splits, the learning of models fails. When we specifically focus on the performance of MM-Net as compared with the baselines, we discover that in 46 out of the 100 splits, the MM-Net outperformed the UME-Net baseline. This observation implies that for certain data splits, the histology images boost the generalization performance of the prediction model, and therefore, its potential utility in preclinical and clinical settings. A further investigation is required to better understand the cases and data characteristics in which histology images improve response prediction.

Technological progress in digital pathology and high-throughput omic profiling have led researchers to generate big data repositories of histology images and omics data, as well as algorithms to jointly analyze these diverse data types. Several papers have explored multimodal architectures that combine histology images with omics data for predicting survival outcomes of cancer patients. Mobadersany et al. demonstrated that a CNN-based supervised learning model combined with cox regression accurately predicts survival outcomes of glioma patients from histology and mutation data (21). Cheerla et al. proposed an unsupervised learning method to learn a low-dimensional representation for each feature type and, consequently, concatenated the learned representations to predict survival outcome of cancer patients (22). They have also demonstrated on 20 cancer types that a custom dropout layer that randomly drops an entire feature vector improves predictions. Building upon existing works, Chen et al. introduced a supervised architecture for multimodal fusion of histology and omics data to predict patient survival and applied their method to glioma and clear cell renal cell carcinoma patients (23). The model uses graph convolutional network (GCN) and CNN to encode histology image data and feed-forward network for mutation data. Each encoded feature vector is passed through an attention mechanism and subsequently fused *via* a Kronecker product. The cox regression is finally used to predict patient survival. While these papers do not consider drug treatments in their analysis, they exploit modern approaches for enhancing predictions of multimodal NNs with histology and omics data and can be further explored for drug response prediction.

A wide spectrum of methods is available in vision applications for inducing changes in images that allow for data augmentation (50). In this study, we exploit the lack of invariance to permutation as the means to augment the sample size. Recently, additional methods have been proposed for augmenting transcriptomic data which can potentially be combined and provide further improvement in predicting drug response (51–53). Considering the scale of existing PDX datasets, data fusion and augmentation provide promising research directions for enhancing predictive capabilities with PDXs. However, special care should be taken because high-dimensional feature sets can often lead to severe overfitting and poor generalization. Presumably to mitigate overfitting, Nguyen et al. (11) and Kim et al. (12) used feature selection methods to reduce the dimensionality of PDX data while considering a single omics feature type at a time. Therefore, multimodal learning exhibits a tradeoff between enriching the feature space *via* multimodal fusion and overfitting. To alleviate this tradeoff, alternative methods can be explored to reduce the feature space while incorporating multiple feature types (54). With the methods proposed in this study and ongoing research into novel augmentation and fusion techniques, PDX pharmaco-omic datasets may become more suitable for modern deep learning techniques and further increase interest for building prediction models to advance precision oncology.

## 5. Conclusions

Deep learning methods have shown promising results in predicting drug response in cancer cell lines. While PDXs are presumed to better mimic human cancer, drug response datasets with this cancer model are substantially smaller as compared to cell line datasets. We investigate multimodal learning and data augmentation methods to address the challenge of limited drug response sample size. Our results suggest that data augmentation and integration of histology images and gene expressions can improve prediction performance of drug response in PDXs.

## Data availability statement

Publicly available datasets were analyzed in this study. This data can be found at: https://pdmr.cancer.gov/database/default.htm.

## Ethics statement

The animal study was reviewed and approved by FNLCR is accredited by the Association for Assessment and Accreditation of Laboratory Animal Care International and follows the Public Health Service Policy for the Care and Use of Laboratory Animals. All studies were conducted according to an approved animal care and use committee protocol in accordance with procedures outlined in the Guide for Care and Use of Laboratory Animals 8th Edition.

## Author contributions

APa, TB, YZ, and RS: conceptualization. APa, YZ, JDol, SK, MS, and YE: data curation. APa and YZ: formal analysis. RS: funding acquisition. APa and YE: investigation and methodology. TB, APe, YE, and RS: project administration and resources. APa, YZ, and JDol: software. YE and JDor: validation. APa: writing—original draft preparation. APa, TB, YZ, JDol, APe, YE, and RS: writing—review and editing. All authors have read and agreed to the published version of the manuscript.
