# Supplementary material for: Data augmentation and multimodal learning for predicting drug response in patient-derived xenografts from gene expressions and histology images
Source: Front Med (Lausanne). 2023 Mar 7;10:1058919. doi: 10.3389/fmed.2023.1058919 (PMC10027779; doi:10.3389/fmed.2023.1058919)
Supplement: Supplementary file 1 [file Data_Sheet_1.PDF]

Supplementary material

Table S1. This table lists performance scores, including MCC (Matthew's correlation coefficient), AUPRC (area under the precision-recall curve), and AUROC (area under the receiver operating characteristic curve) of the drug response prediction models (UME-Net, UME-Net$_{org}$, UME-Net$_{pairs}$, UMH-Net, MM-Net, and LGBT). The differences between the different models are described in the text and Table 2 of the main manuscript. To compute the average score for each metric and model, the predictions were aggregated via mean across the data splits. In addition, for each metric and pair of models, we compute the pair t-test and report the p-values to assess statistical significance of the means.

	Paired t-test			Wilcoxon signed-rank test		
Compared models	AUPRC	AUROC	MCC	AUPRC	AUROC	MCC
MM-Net vs UMH-Net	0	0.9973	0	0	0.8056	0
MM-Net vs UME-Net	0.8247	0.2374	0.261	0.7596	0.2798	0.1938
MM-Net vs LGBM	0.1873	0.414	0.01	0.1799	0.3444	0.0215
MM-Net vs UME-Net_org	0.0012	0.0137	0	0.0018	0.0068	0.0002
MM-Net vs UME-Net_pairs	0.0001	0	0	0	0	0
UMH-Net vs UME-Net	0	0.4204	0	0	0.4221	0
UMH-Net vs LGBM	0.0019	0.4017	0.0365	0.0175	0.2437	0.0649
UMH-Net vs UME-Net_org	0.0189	0.0268	0.188	0.0907	0.0413	0.2078
UMH-Net vs UME-Net_pairs	0.7303	0	0.6566	0.3677	0	0.7774
UME-Net vs LGBM	0.1491	0.8464	0.0692	0.2058	0.9249	0.1047
UME-Net vs UME-Net_org	0.0037	0.0008	0.0007	0.0004	0.0004	0.0016
UME-Net vs UME-Net_pairs	0.0001	0	0	0	0	0
LGBM vs UME-Net_org	0.2251	0.0047	0.3331	0.1032	0.0012	0.1907
LGBM vs UME-Net_pairs	0.018	0	0.0181	0.0222	0	0.0361
UME-Net_org vs UME-Net_pairs	0.1103	0.009	0.0867	0.0716	0.0132	0.0756
MM-Net vs UMH-Net	0	0.9973	0	0	0.8056	0

Below are the AUROC plots for the 100 data splits

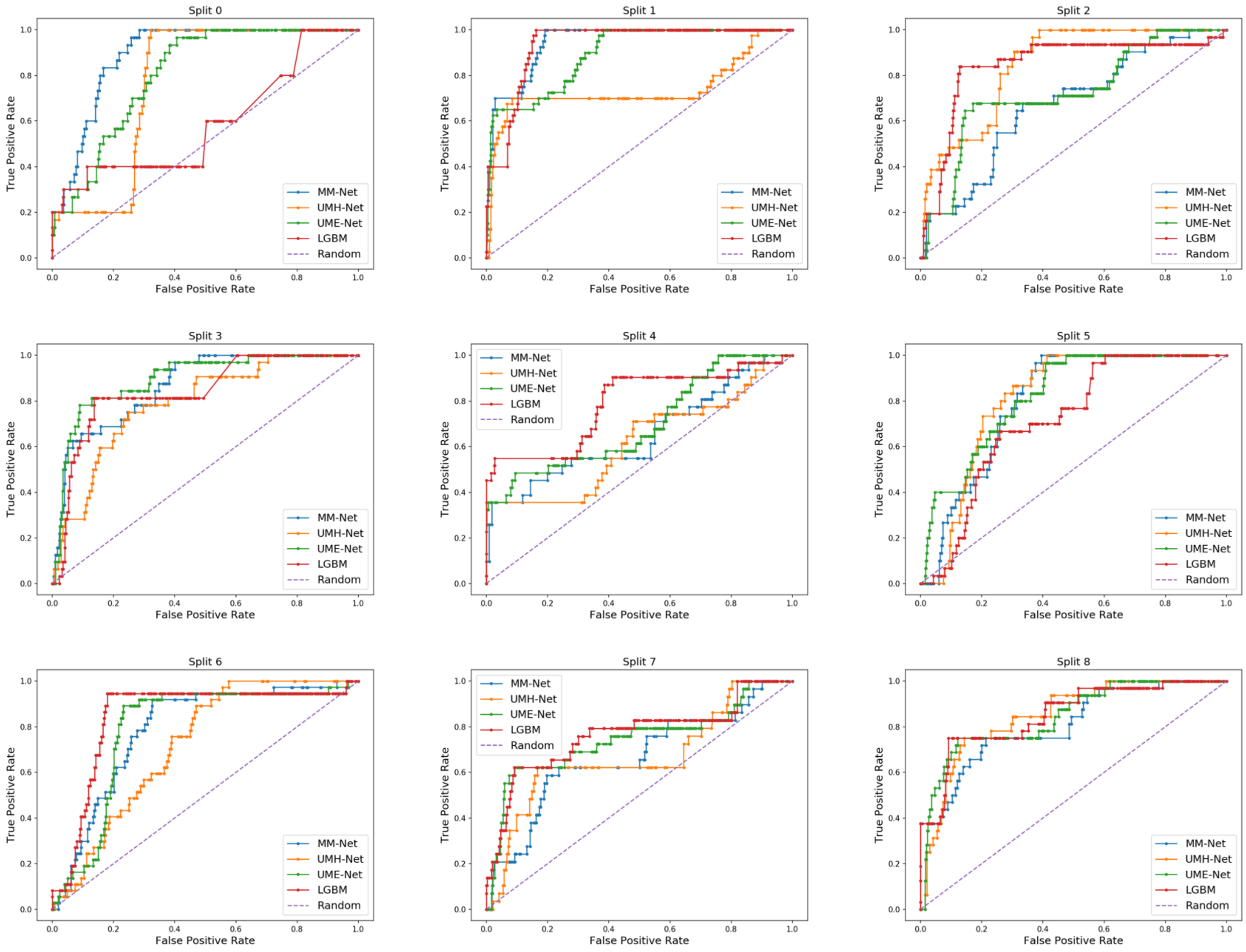

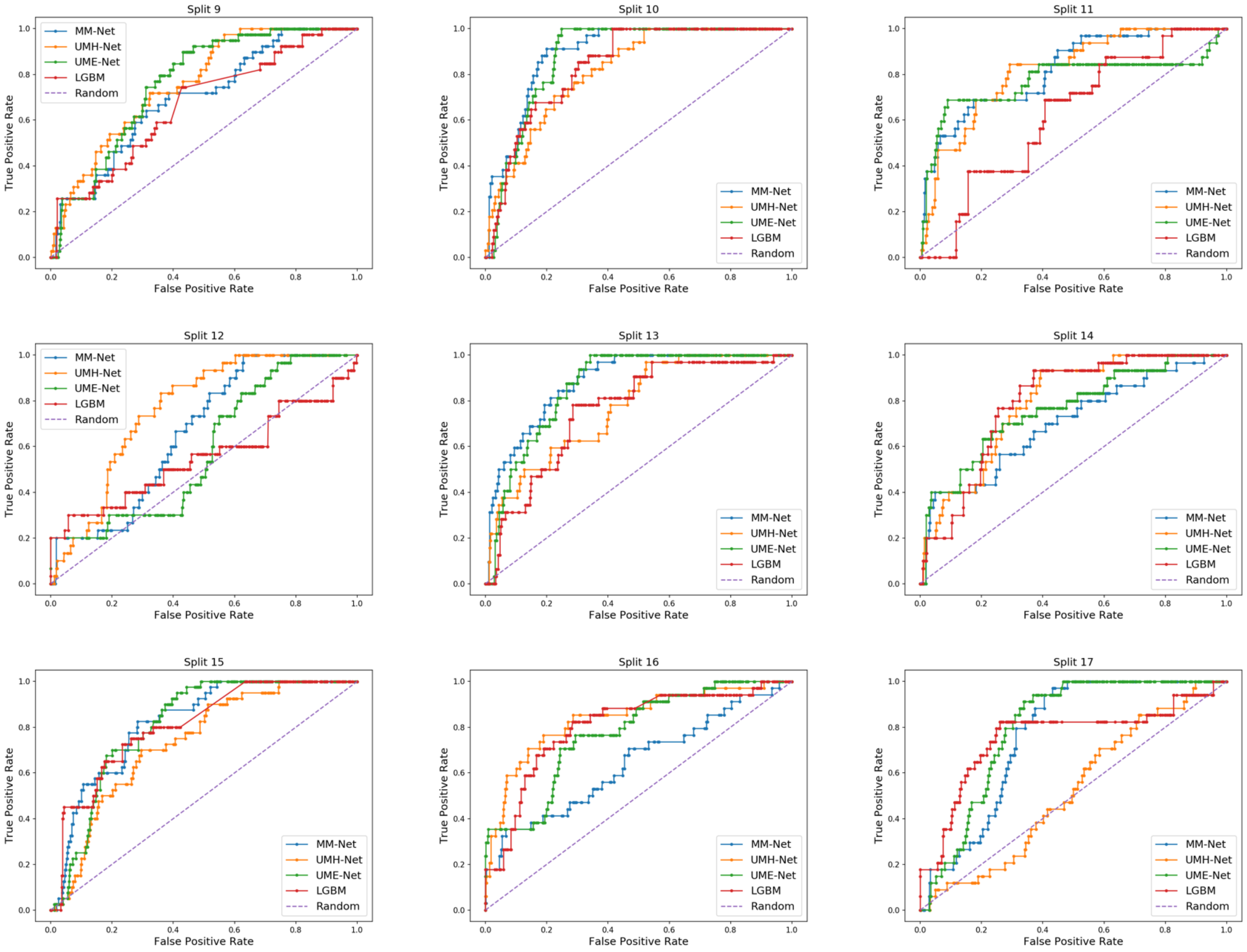
Split 9
Split 10
Split 11
Split 12
Split 13
Split 14
Split 15
Split 16
Split 17
True Positive Rate
False Positive Rate
MM-Net
UMH-Net
UME-Net
LGBM
Random
0.0
0.2
0.4
0.6
0.8
1.0

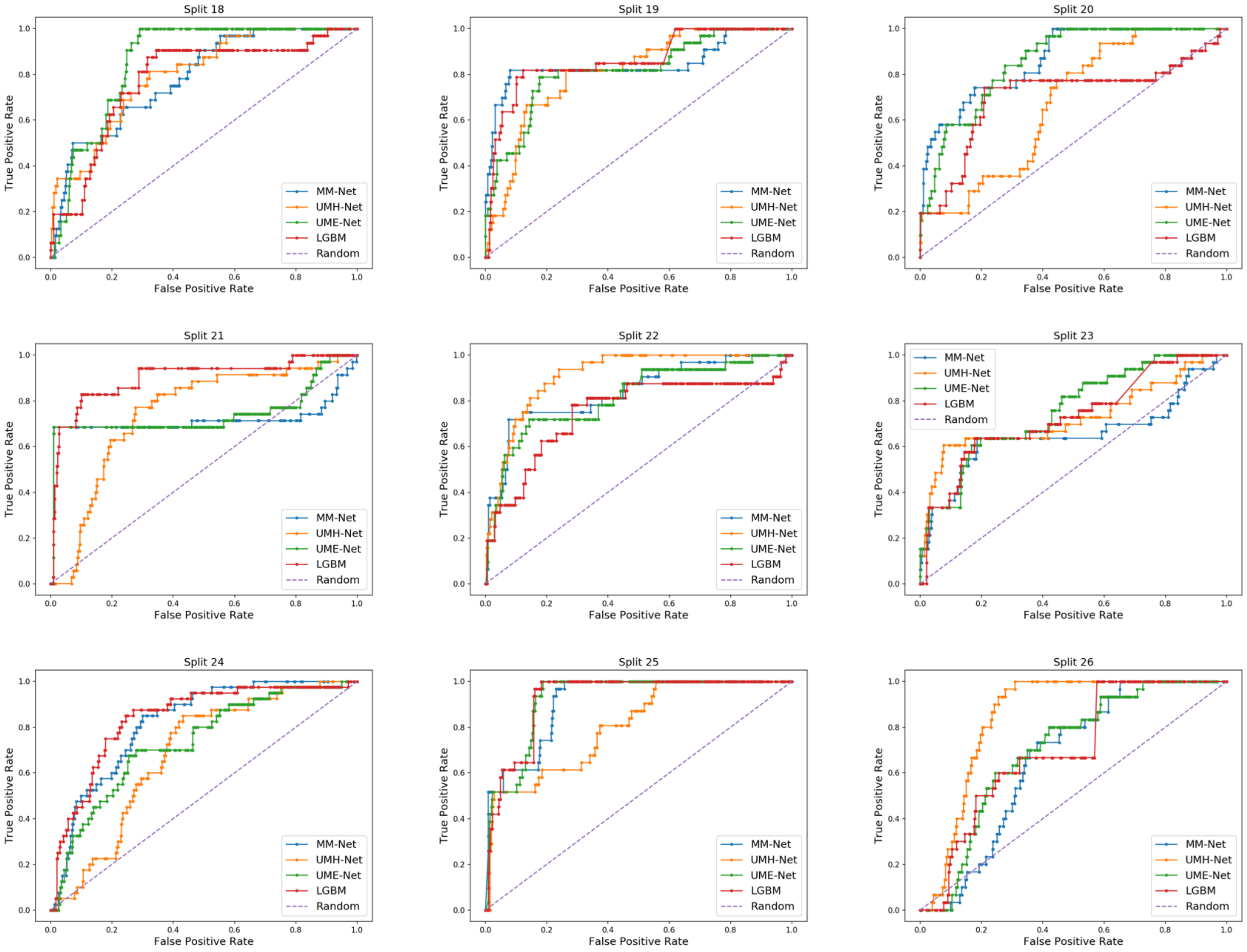
Split 18
Split 19
Split 20
Split 21
Split 22
Split 23
Split 24
Split 25
Split 26
True Positive Rate
False Positive Rate
MM-Net
UMH-Net
UME-Net
LGBM
Random

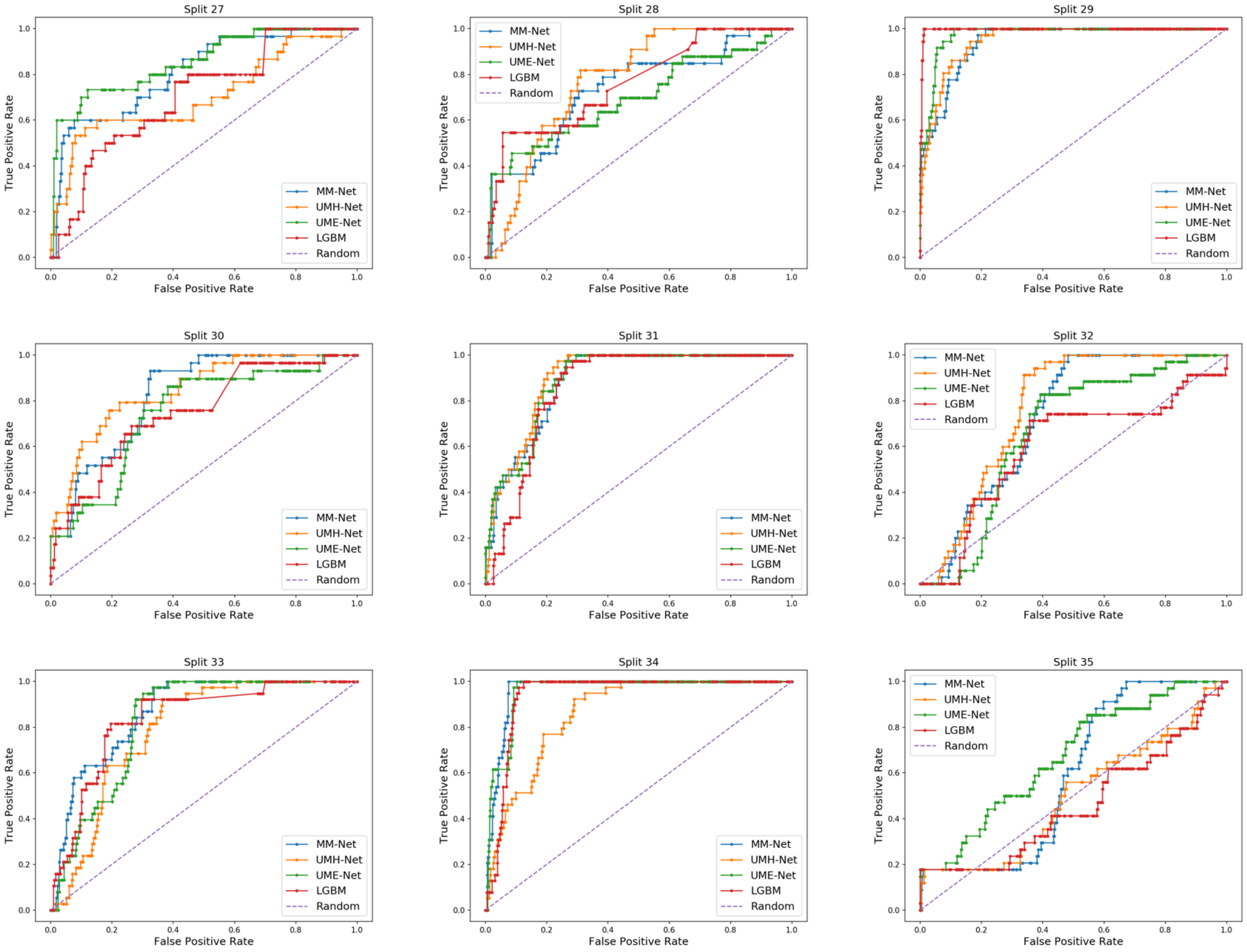
Split 27
Split 28
Split 29
Split 30
Split 31
Split 32
Split 33
Split 34
Split 35
True Positive Rate
False Positive Rate
0.0
0.2
0.4
0.6
0.8
1.0
MM-Net
UMH-Net
UME-Net
LGBM
Random

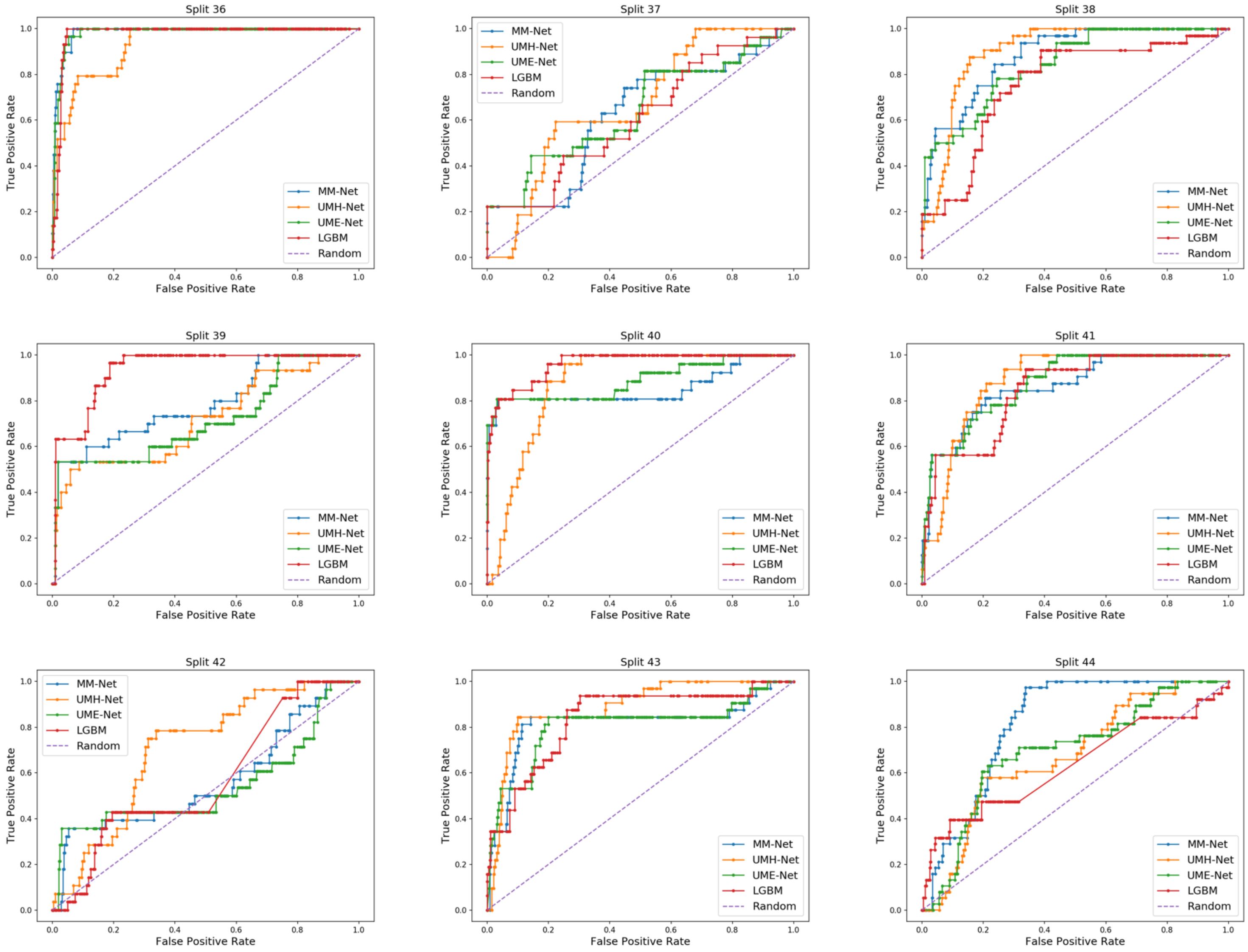

Split 36
Split 37
Split 38
Split 39
Split 40
Split 41
Split 42
Split 43
Split 44
True Positive Rate
False Positive Rate
MM-Net
UMH-Net
UME-Net
LGBM
Random

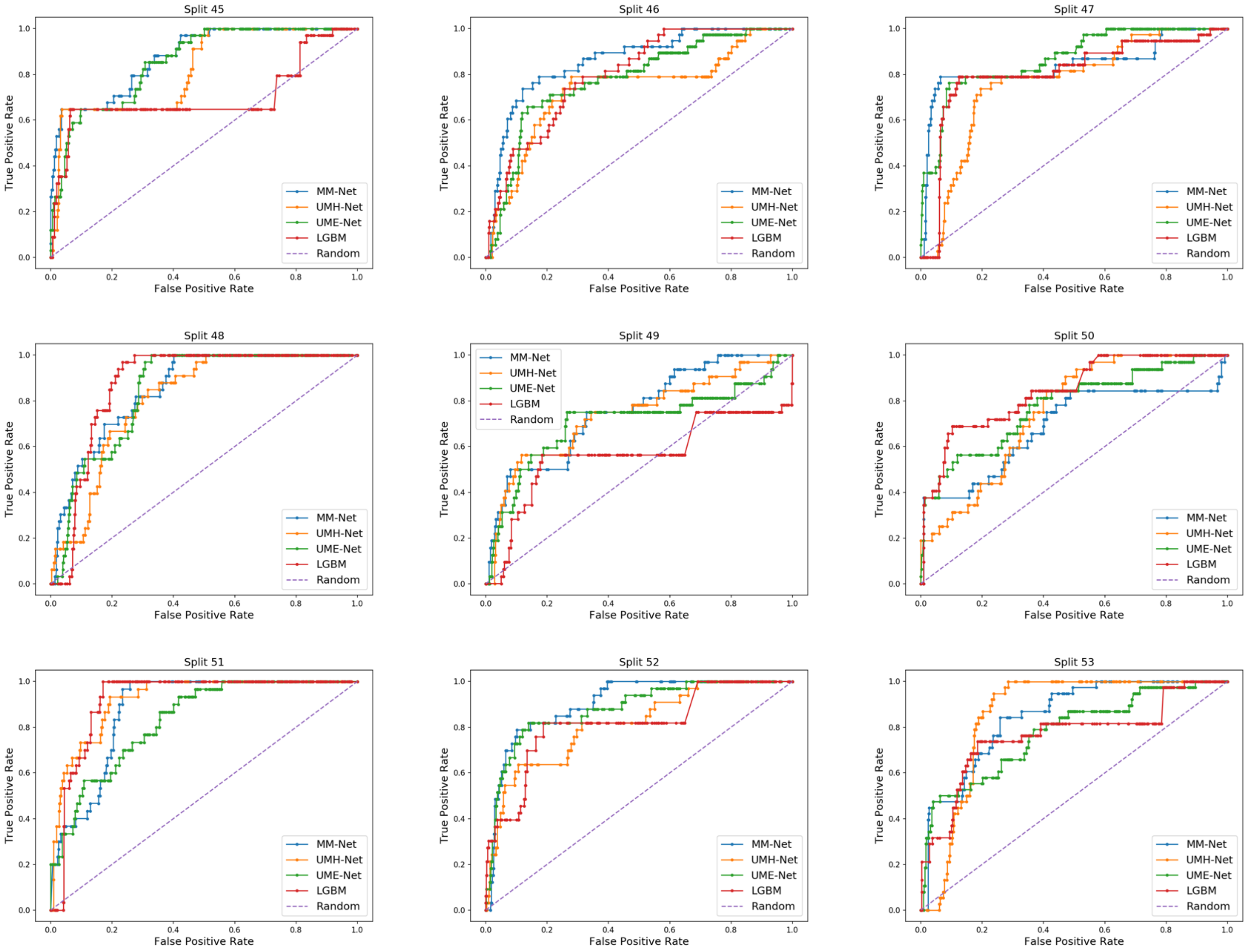

Split 45
Split 46
Split 47
Split 48
Split 49
Split 50
Split 51
Split 52
Split 53
True Positive Rate
False Positive Rate
0.0
0.2
0.4
0.6
0.8
1.0
MM-Net
UMH-Net
UME-Net
LGBM
Random

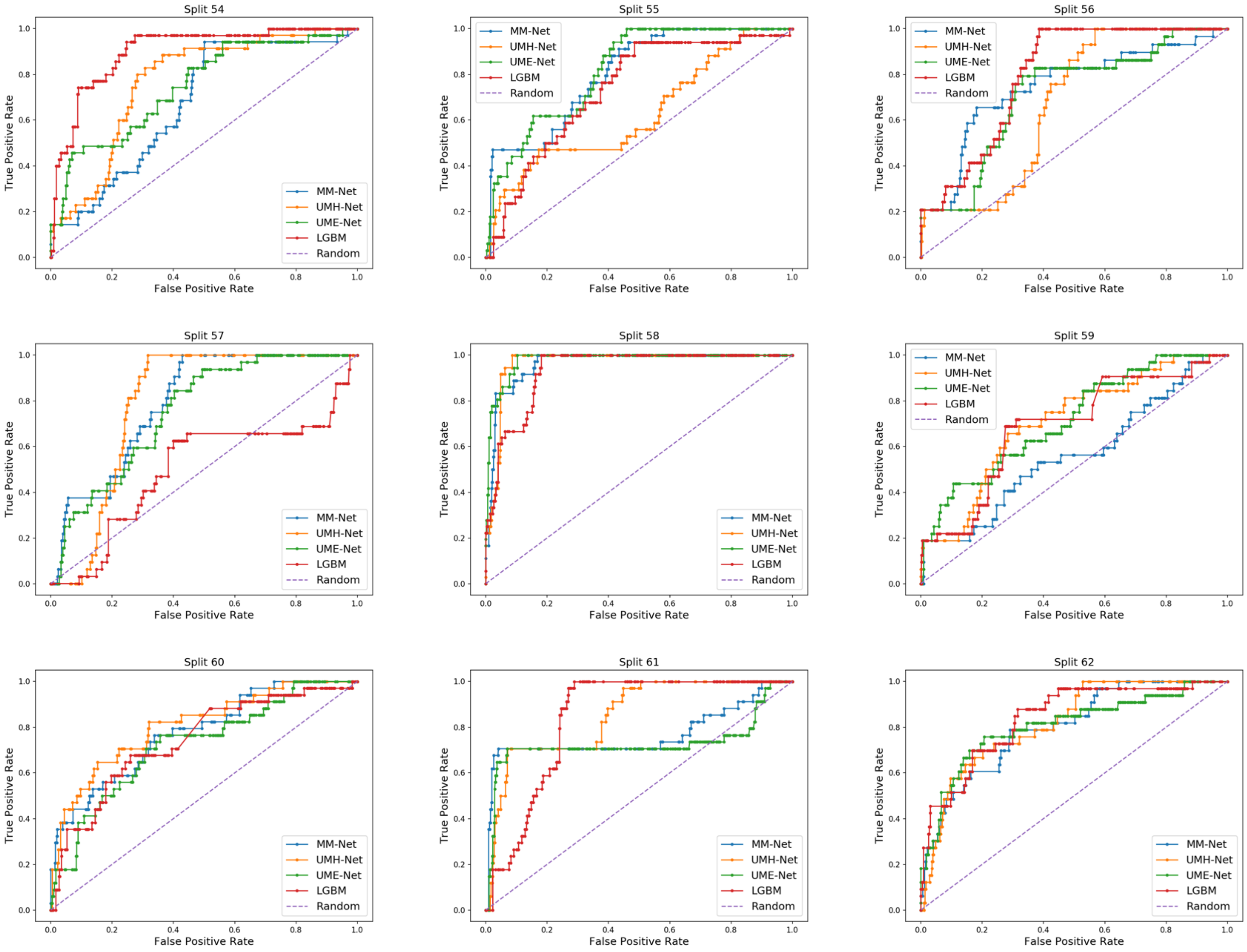

Split 54
Split 55
Split 56
Split 57
Split 58
Split 59
Split 60
Split 61
Split 62
True Positive Rate
False Positive Rate
MM-Net
UMH-Net
UME-Net
LGBM
Random

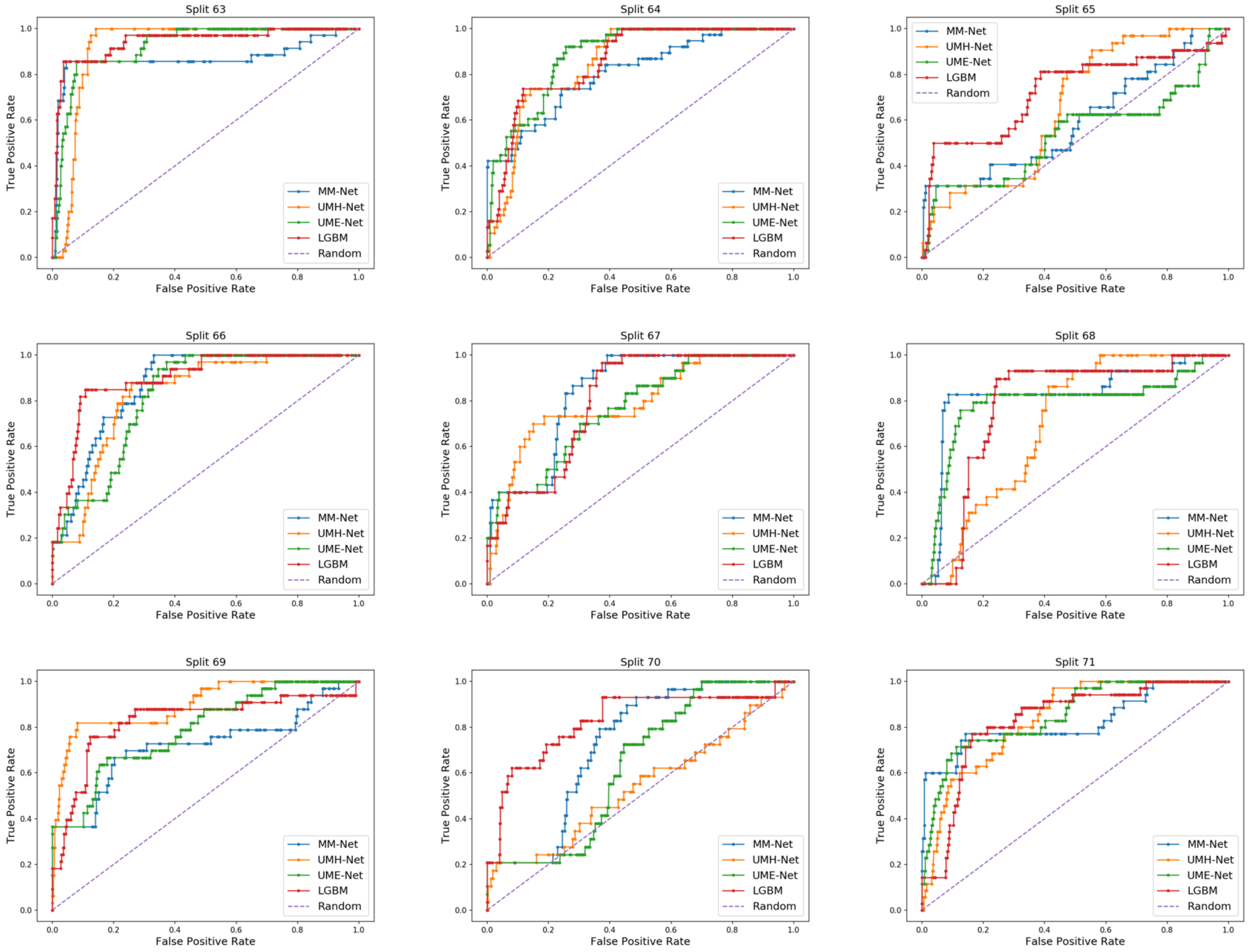
Split 63
Split 64
Split 65
Split 66
Split 67
Split 68
Split 69
Split 70
Split 71
True Positive Rate
False Positive Rate
MM-Net
UMH-Net
UME-Net
LGBM
Random

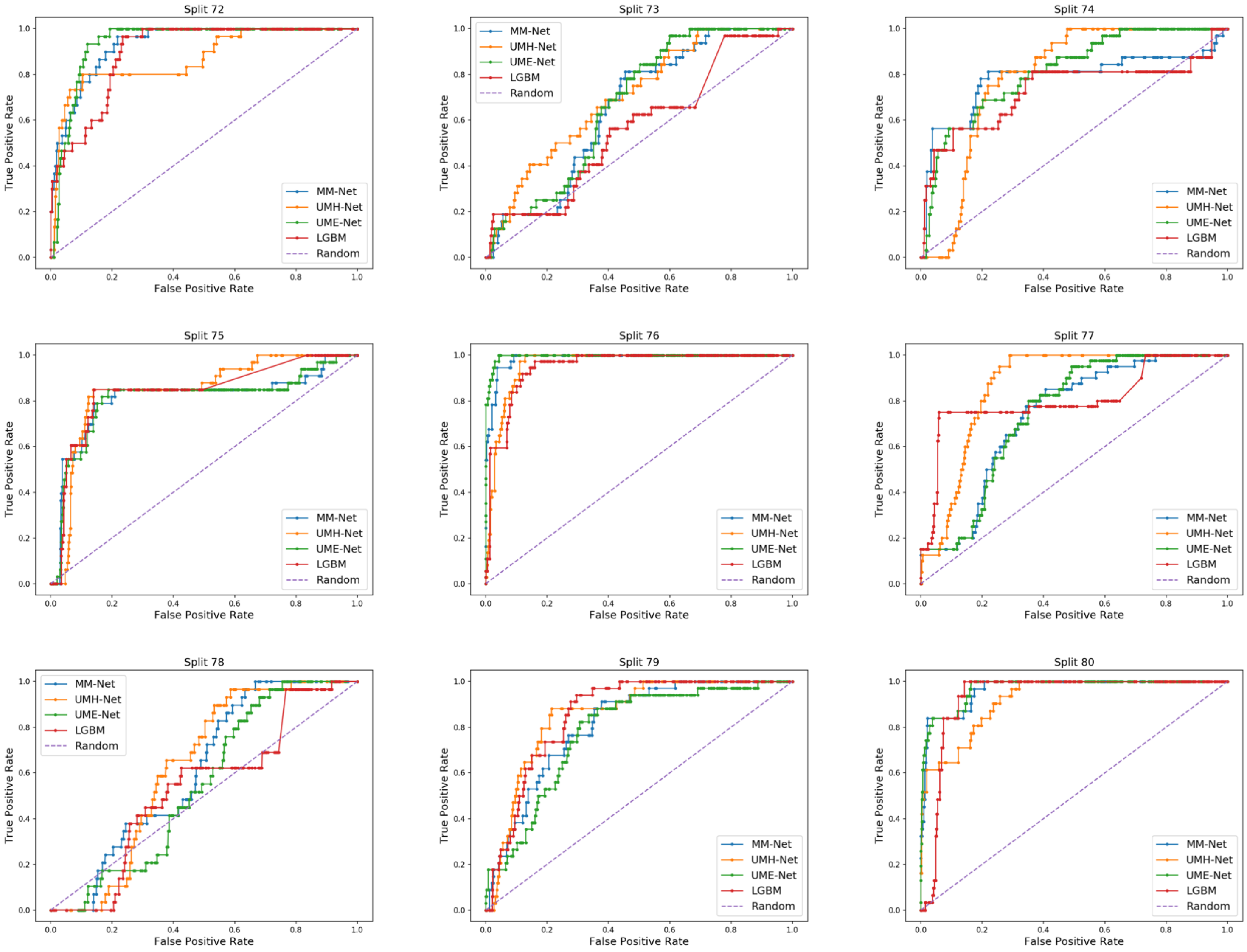

Split 72
Split 73
Split 74
Split 75
Split 76
Split 77
Split 78
Split 79
Split 80
True Positive Rate
False Positive Rate
0.0
0.2
0.4
0.6
0.8
1.0
MM-Net
UMH-Net
UME-Net
LGBM
Random

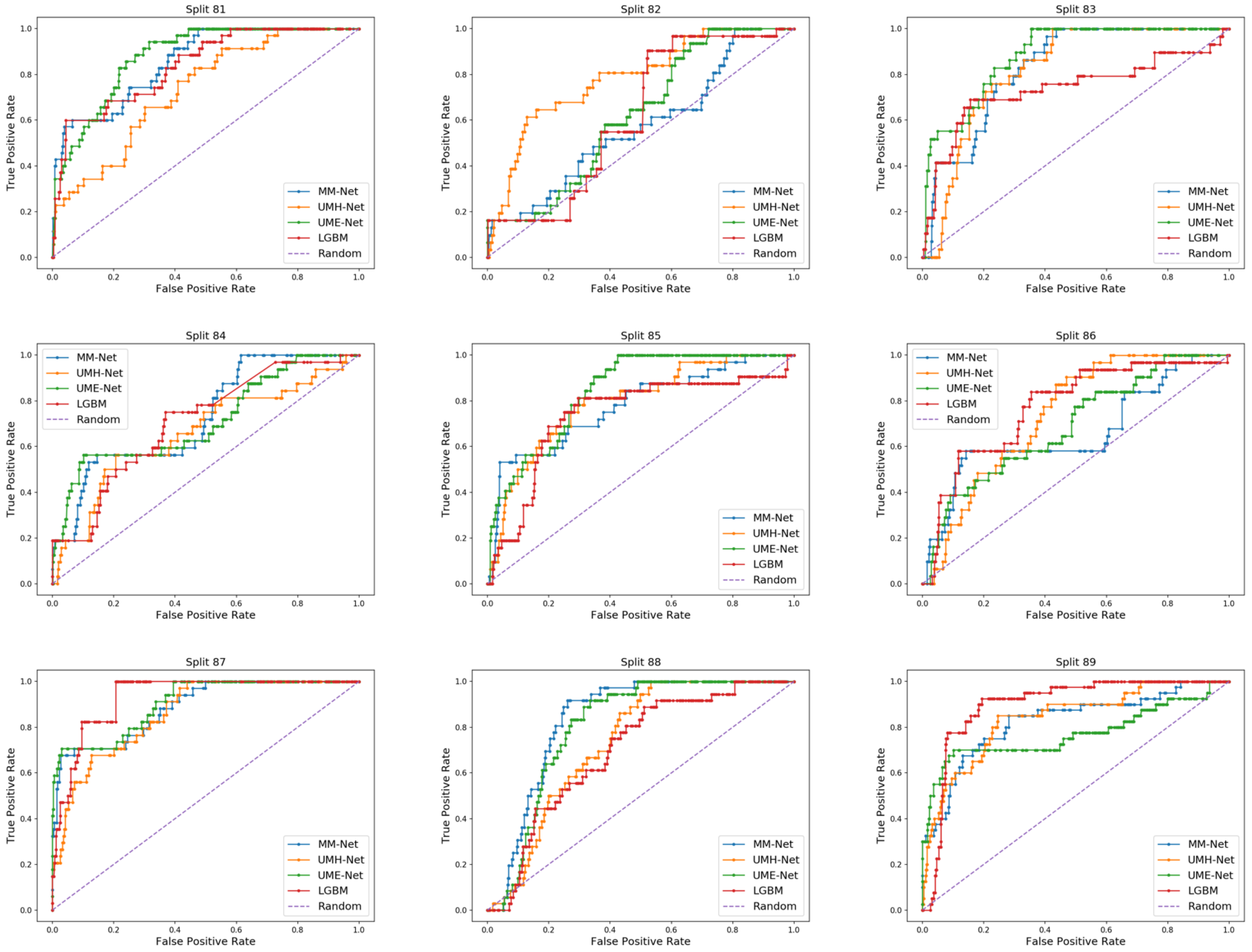

Split 81
True Positive Rate
False Positive Rate
MM-Net
UMH-Net
UME-Net
LGBM
Random
Split 82
True Positive Rate
False Positive Rate
MM-Net
UMH-Net
UME-Net
LGBM
Random
Split 83
True Positive Rate
False Positive Rate
MM-Net
UMH-Net
UME-Net
LGBM
Random
Split 84
True Positive Rate
False Positive Rate
MM-Net
UMH-Net
UME-Net
LGBM
Random
Split 85
True Positive Rate
False Positive Rate
MM-Net
UMH-Net
UME-Net
LGBM
Random
Split 86
True Positive Rate
False Positive Rate
MM-Net
UMH-Net
UME-Net
LGBM
Random
Split 87
True Positive Rate
False Positive Rate
MM-Net
UMH-Net
UME-Net
LGBM
Random
Split 88
True Positive Rate
False Positive Rate
MM-Net
UMH-Net
UME-Net
LGBM
Random
Split 89
True Positive Rate
False Positive Rate
MM-Net
UMH-Net
UME-Net
LGBM
Random

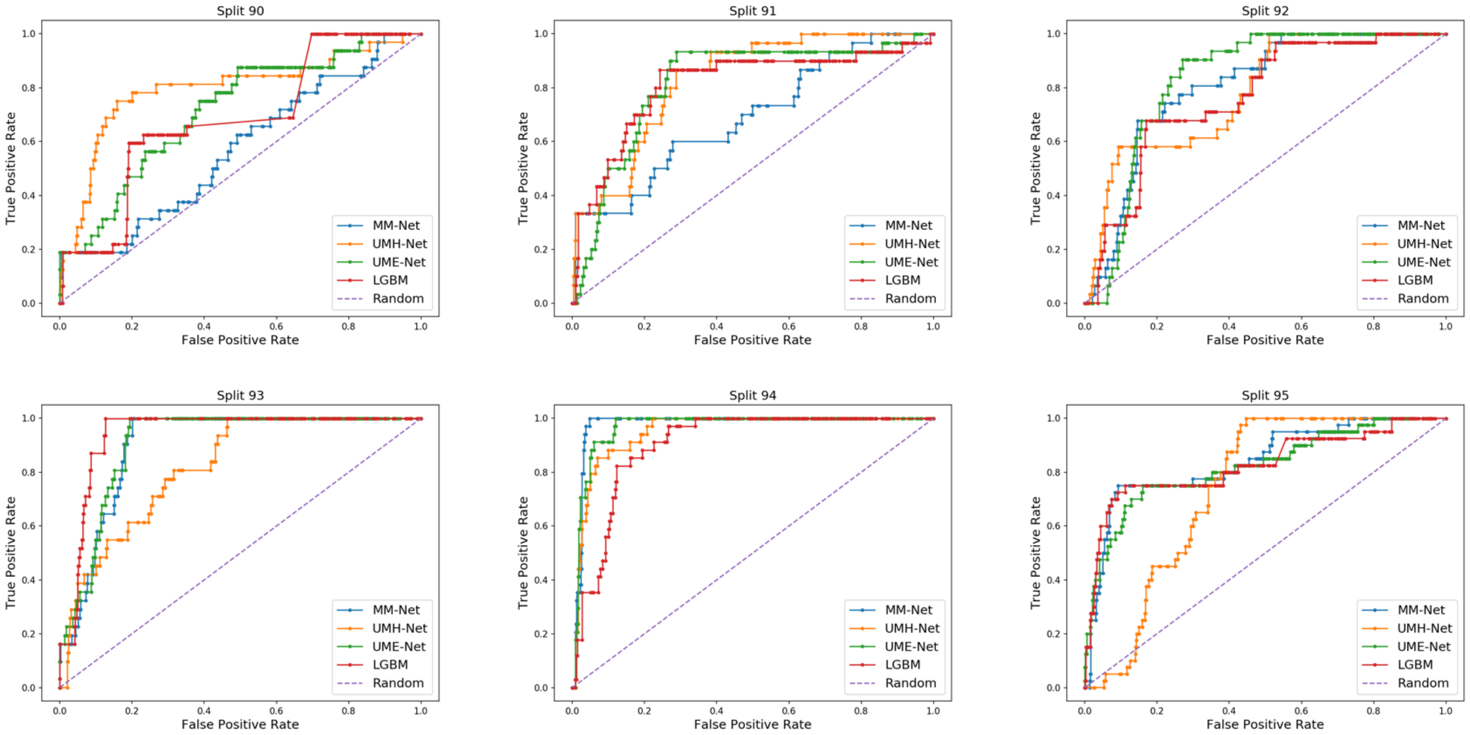
Split 90
Split 91
Split 92
Split 93
Split 94
Split 95
True Positive Rate
False Positive Rate
0.0
0.2
0.4
0.6
0.8
1.0
MM-Net
UMH-Net
UME-Net
LGBM
Random

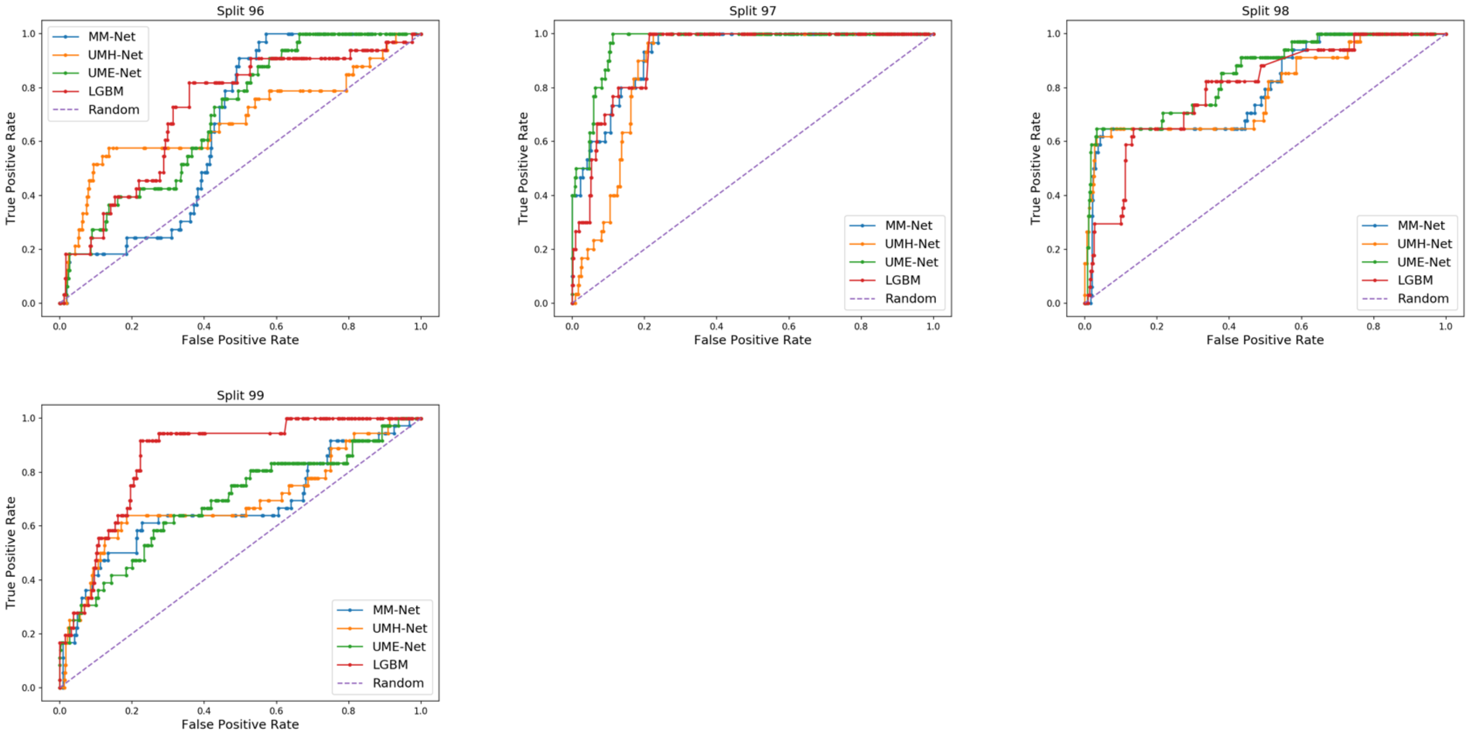
Split 96
Split 97
Split 98
Split 99
True Positive Rate
False Positive Rate
0.0
0.2
0.4
0.6
0.8
1.0
MM-Net
UMH-Net
UME-Net
LGBM
Random

Below are the AUPRC plots for the 100 data splits

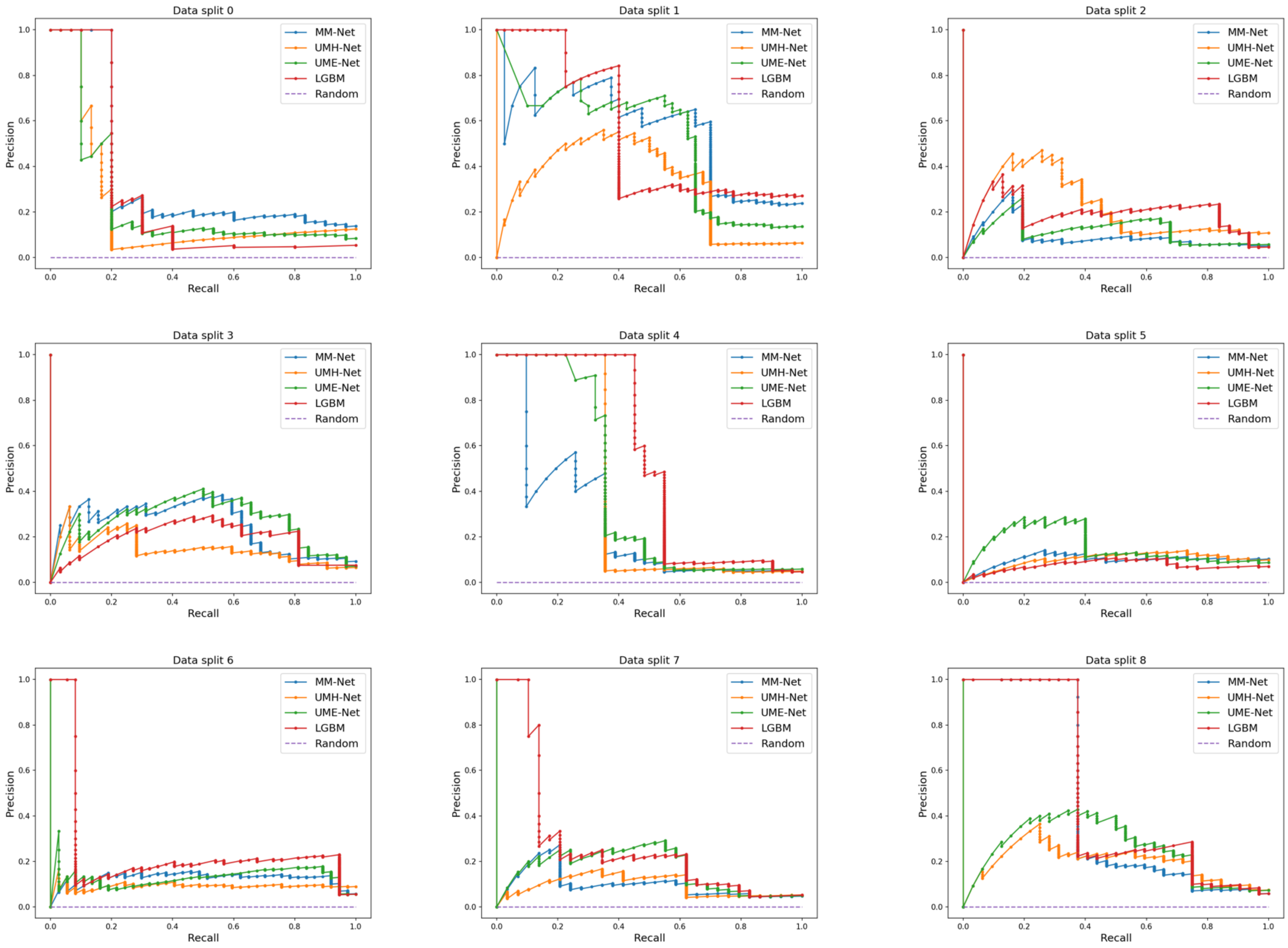

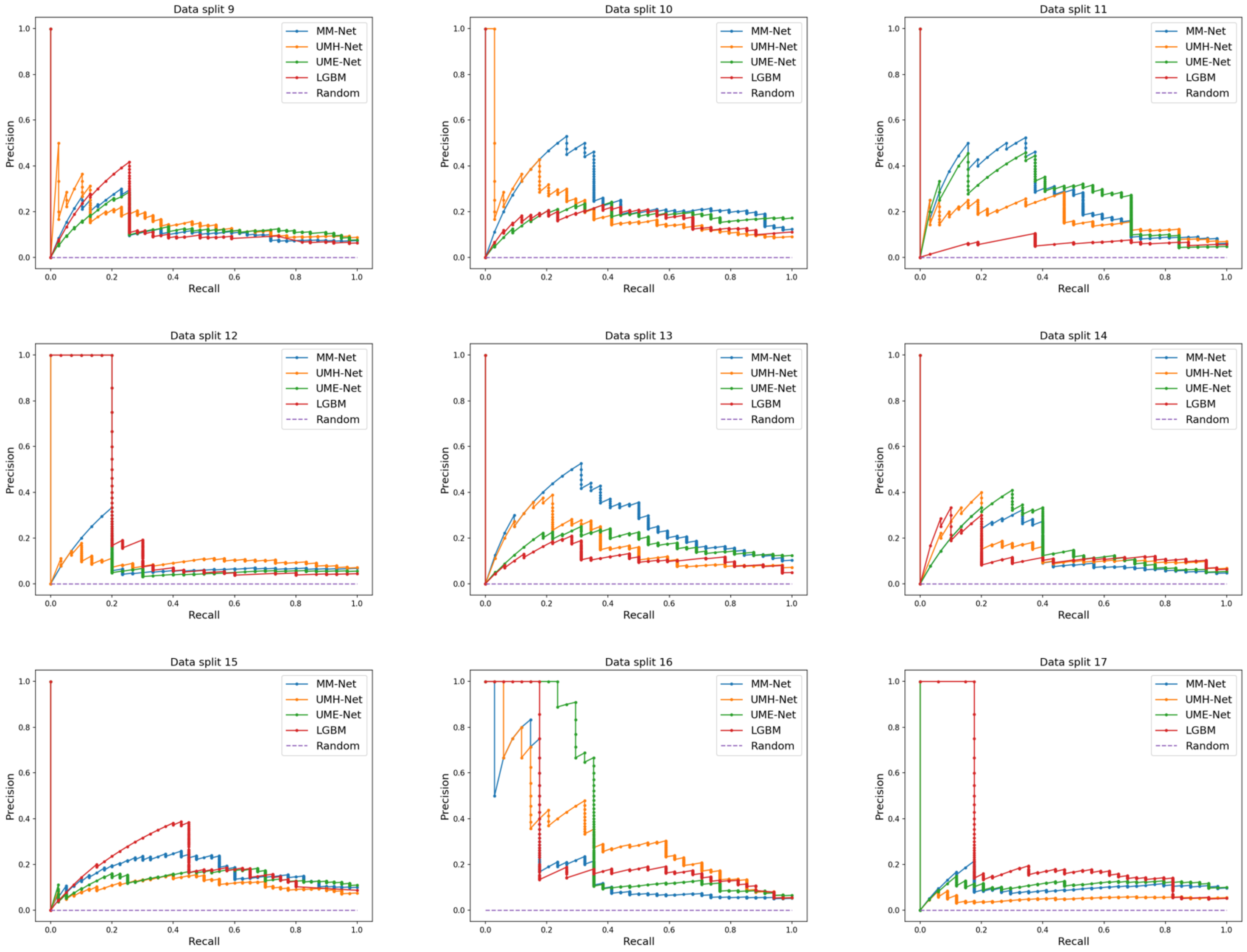
Data split 9
Data split 10
Data split 11
Data split 12
Data split 13
Data split 14
Data split 15
Data split 16
Data split 17
MM-Net
UMH-Net
UME-Net
LGBM
Random
Precision
Recall

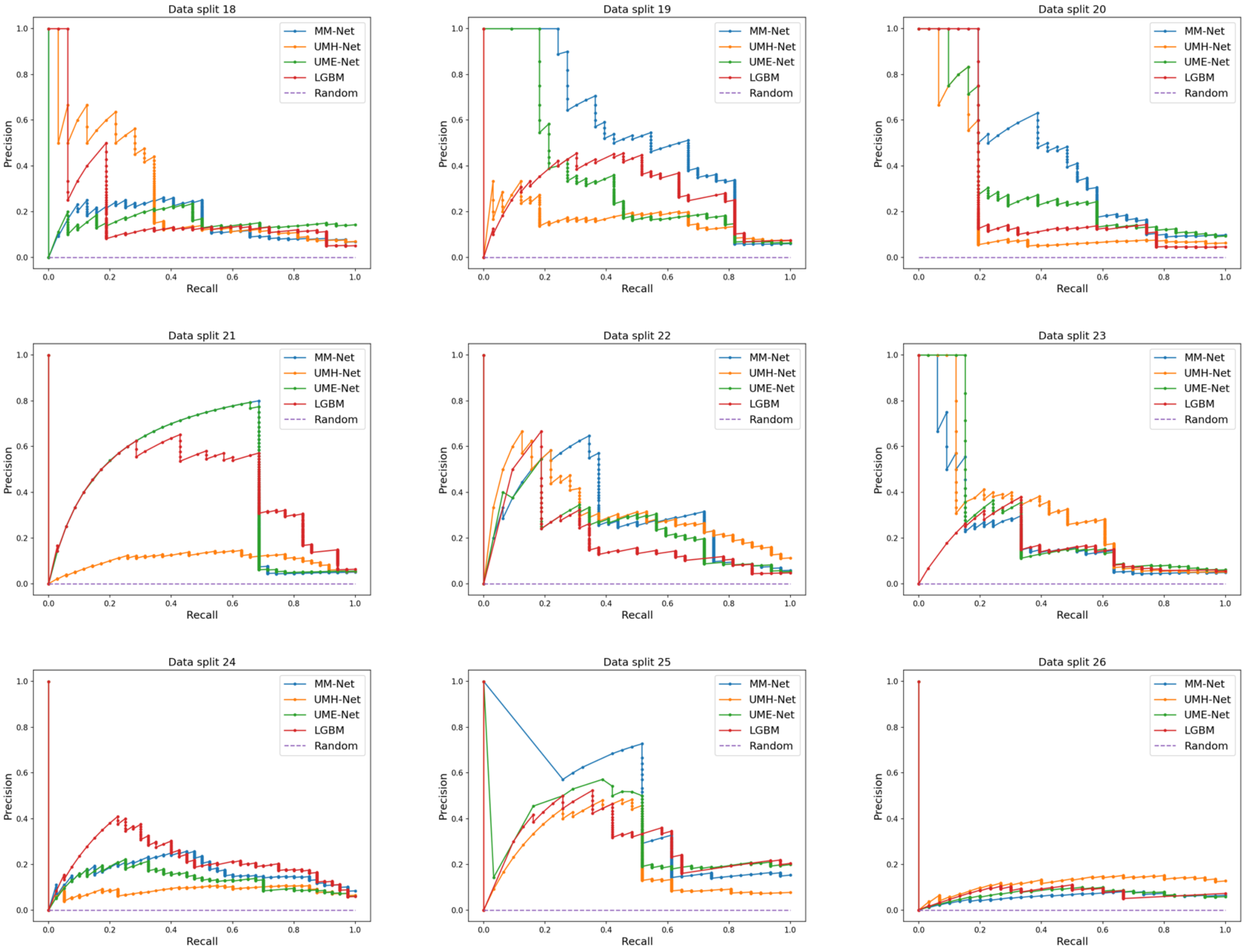
Data split 18
Data split 19
Data split 20
Data split 21
Data split 22
Data split 23
Data split 24
Data split 25
Data split 26
MM-Net
UMH-Net
UME-Net
LGBM
Random
Precision
Recall
0.0
0.2
0.4
0.6
0.8
1.0

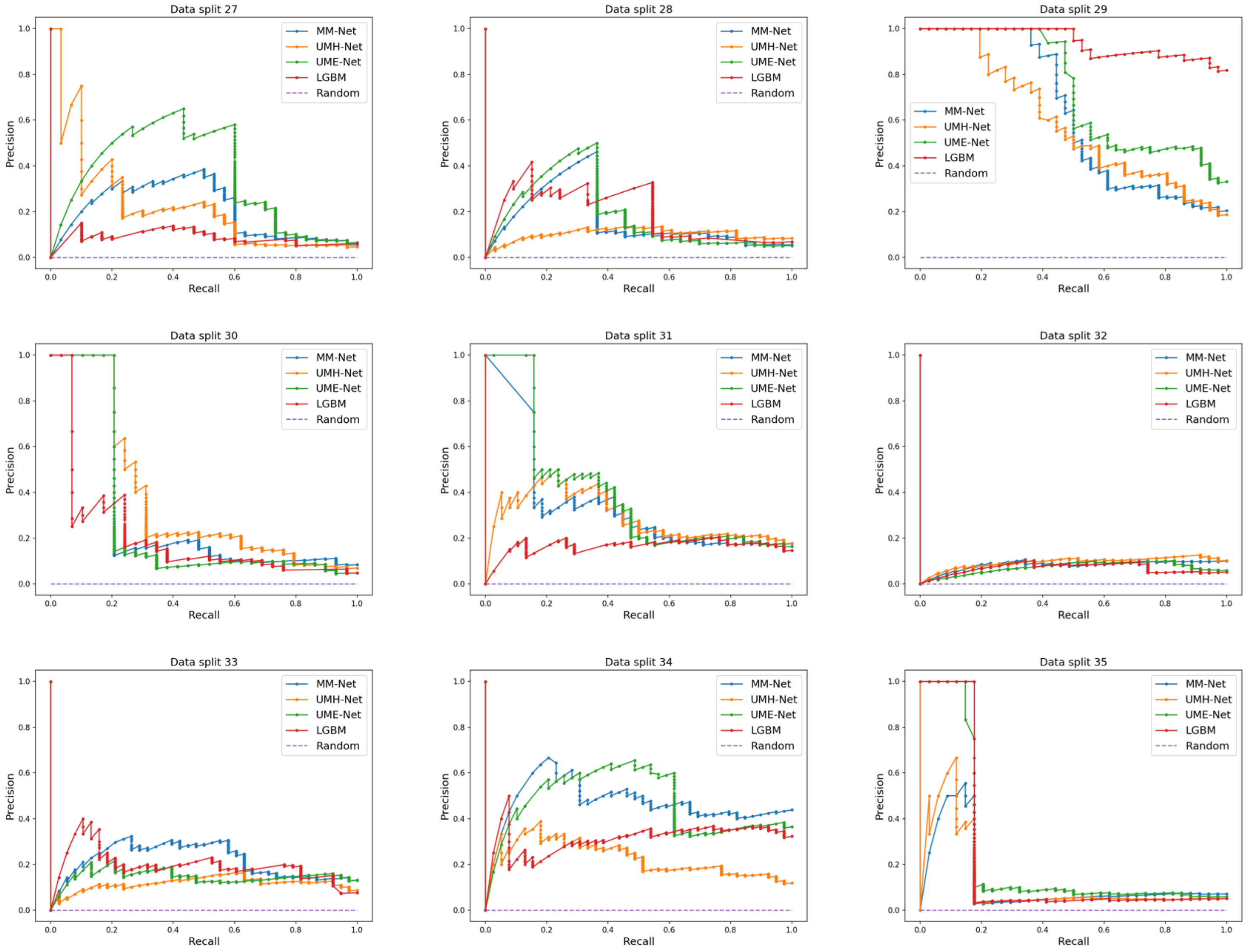

Data split 27
Data split 28
Data split 29
Data split 30
Data split 31
Data split 32
Data split 33
Data split 34
Data split 35
MM-Net
UMH-Net
UME-Net
LGBM
Random
Precision
Recall
0.0
0.2
0.4
0.6
0.8
1.0

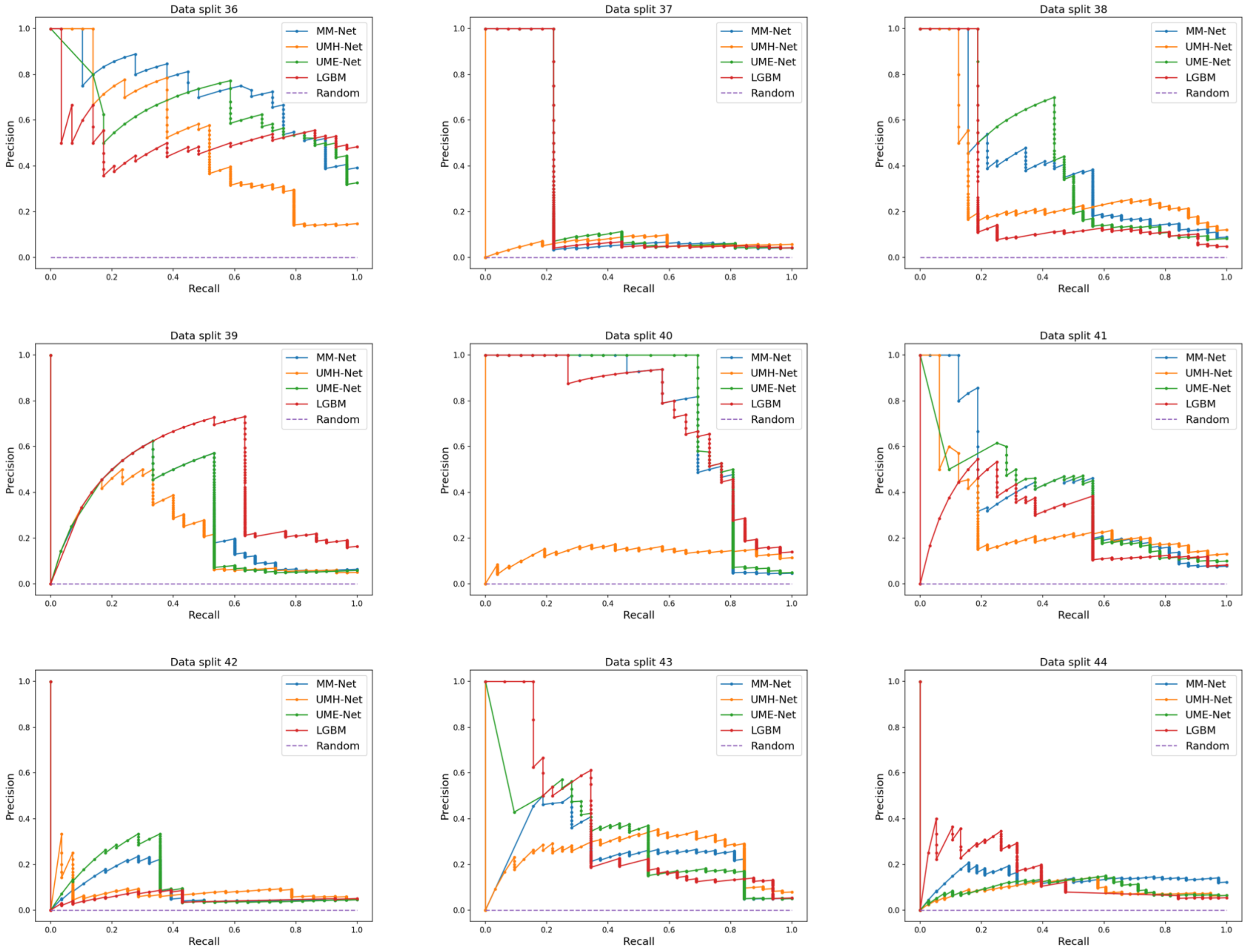
Data split 36
Data split 37
Data split 38
Data split 39
Data split 40
Data split 41
Data split 42
Data split 43
Data split 44
MM-Net
UMH-Net
UME-Net
LGBM
Random
Precision
Recall
0.0
0.2
0.4
0.6
0.8
1.0

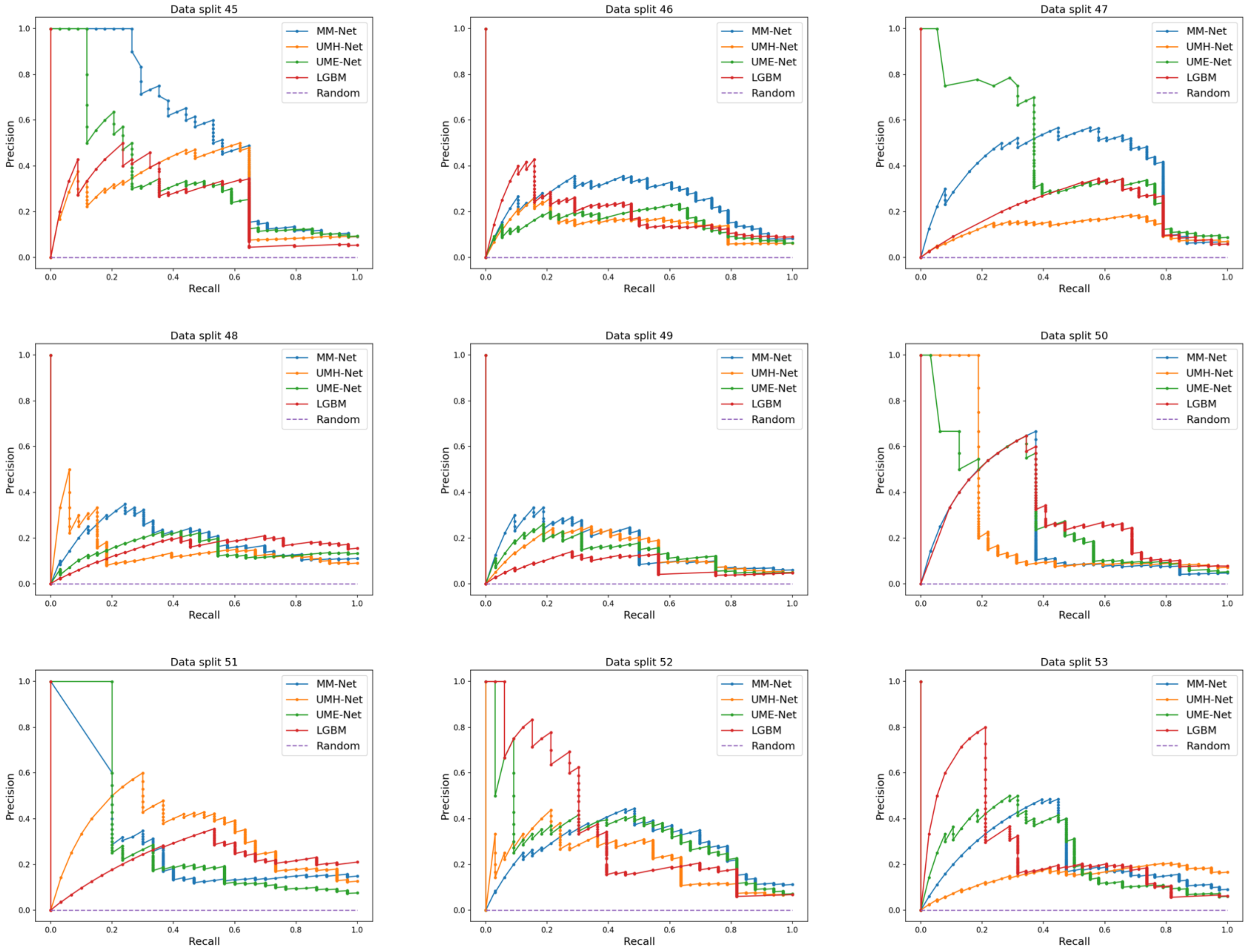
Data split 45
Data split 46
Data split 47
Data split 48
Data split 49
Data split 50
Data split 51
Data split 52
Data split 53
MM-Net
UMH-Net
UME-Net
LGBM
Random
Precision
Recall

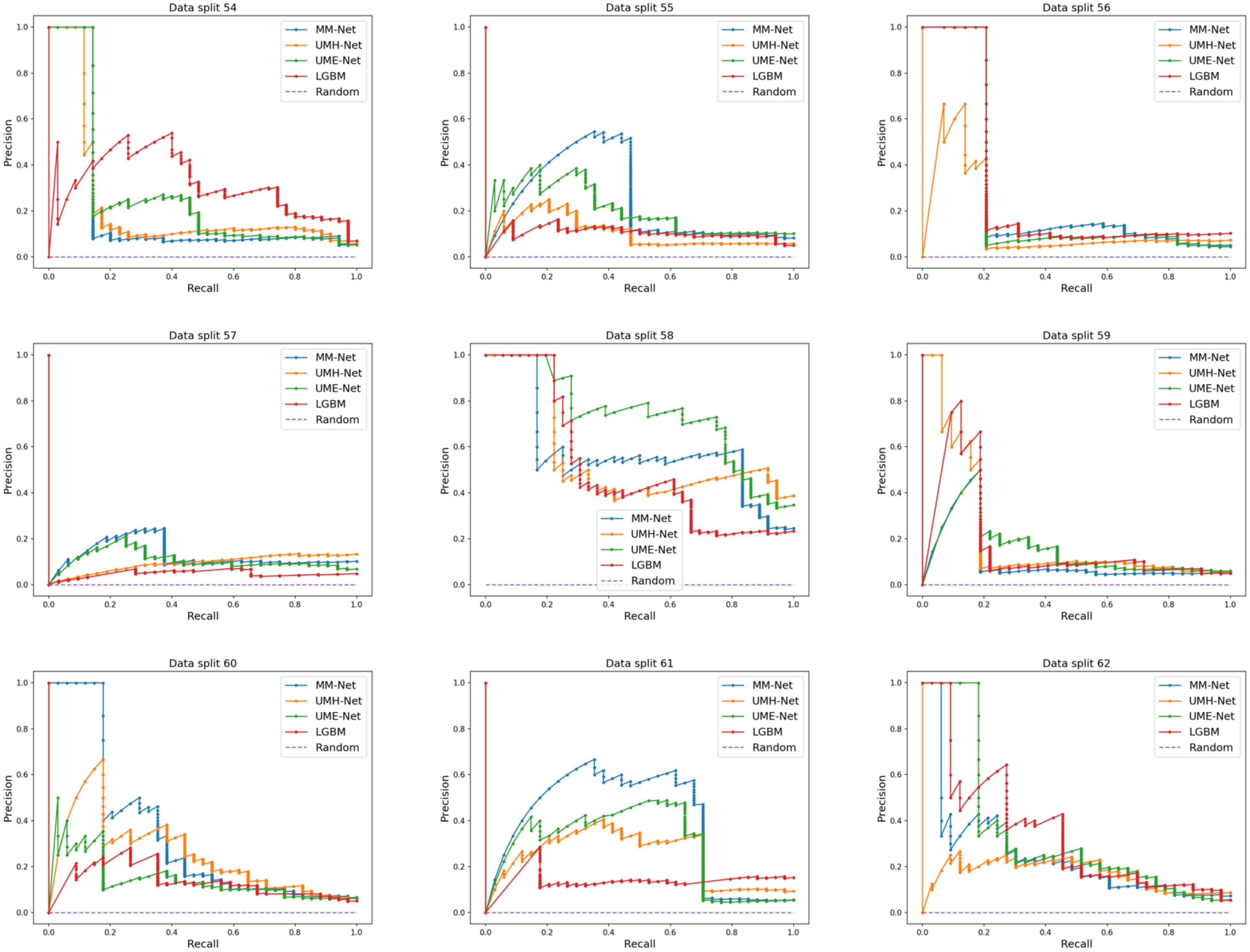

Data split 54
Data split 55
Data split 56
Data split 57
Data split 58
Data split 59
Data split 60
Data split 61
Data split 62
MM-Net
UMH-Net
UME-Net
LGBM
Random
Precision
Recall
0.0
0.2
0.4
0.6
0.8
1.0

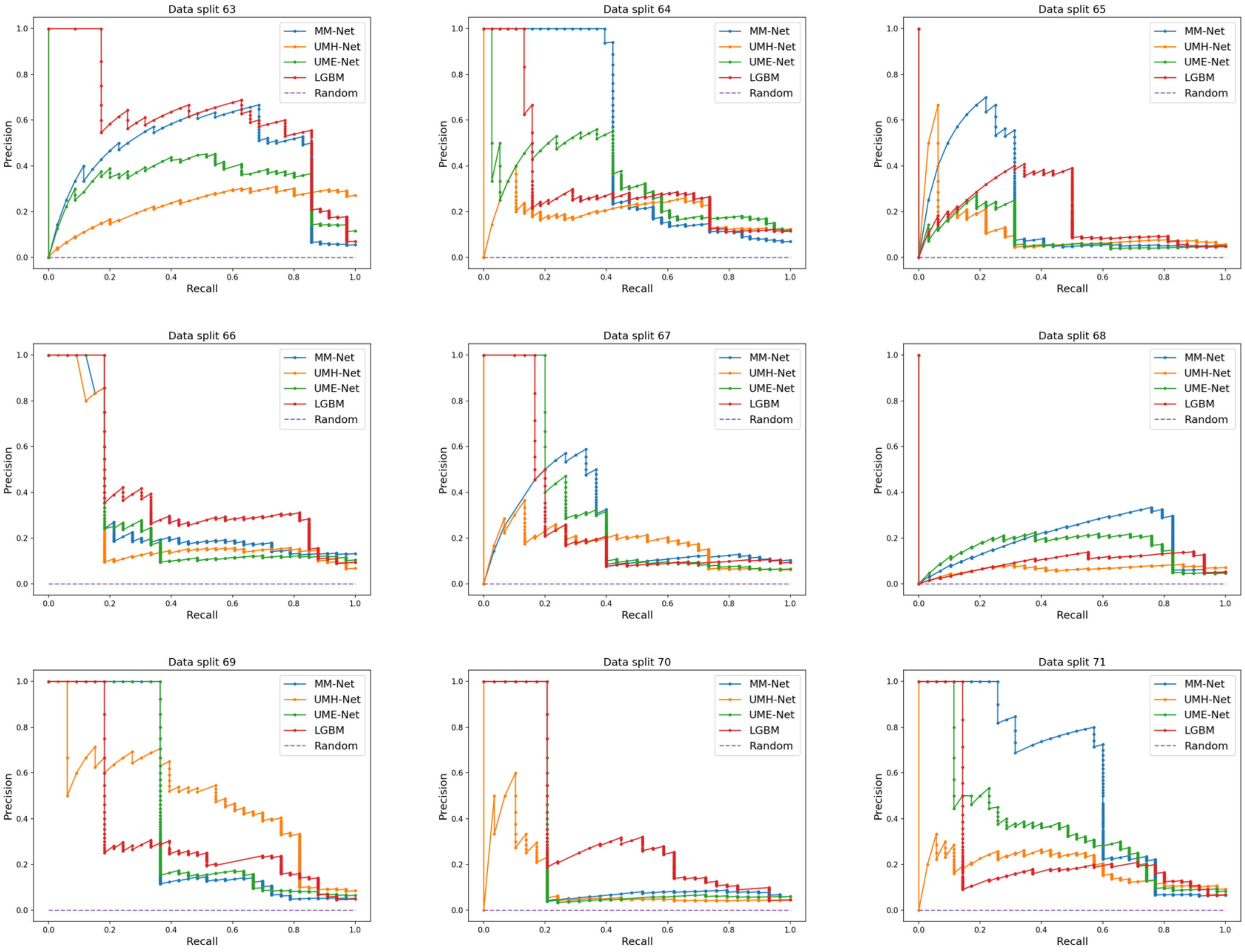
Data split 63
Data split 64
Data split 65
Data split 66
Data split 67
Data split 68
Data split 69
Data split 70
Data split 71
Precision
Recall
MM-Net
UMH-Net
UME-Net
LGBM
Random

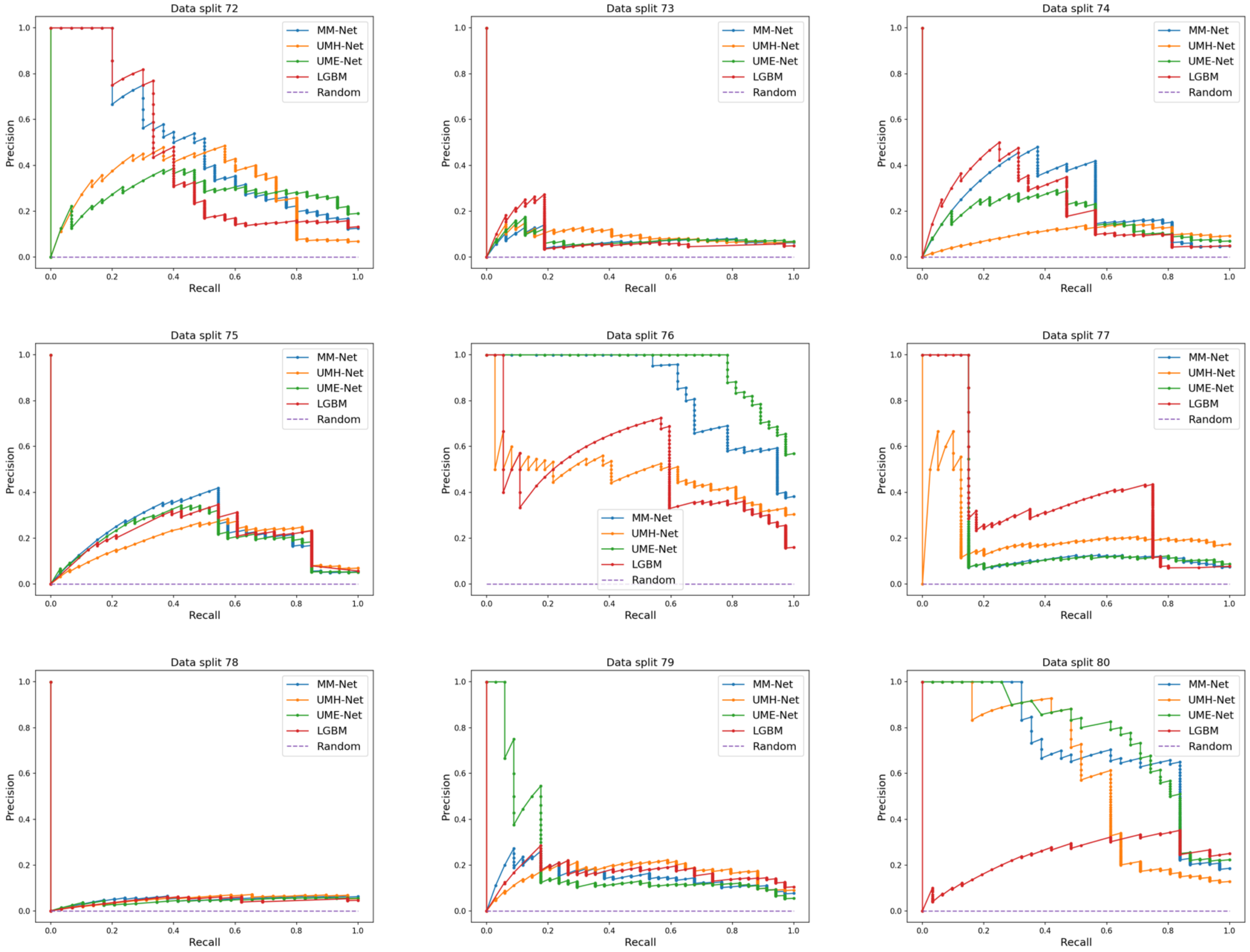
Data split 72
Data split 73
Data split 74
Data split 75
Data split 76
Data split 77
Data split 78
Data split 79
Data split 80
MM-Net
UMH-Net
UME-Net
LGBM
Random
Precision
Recall
0.0
0.2
0.4
0.6
0.8
1.0

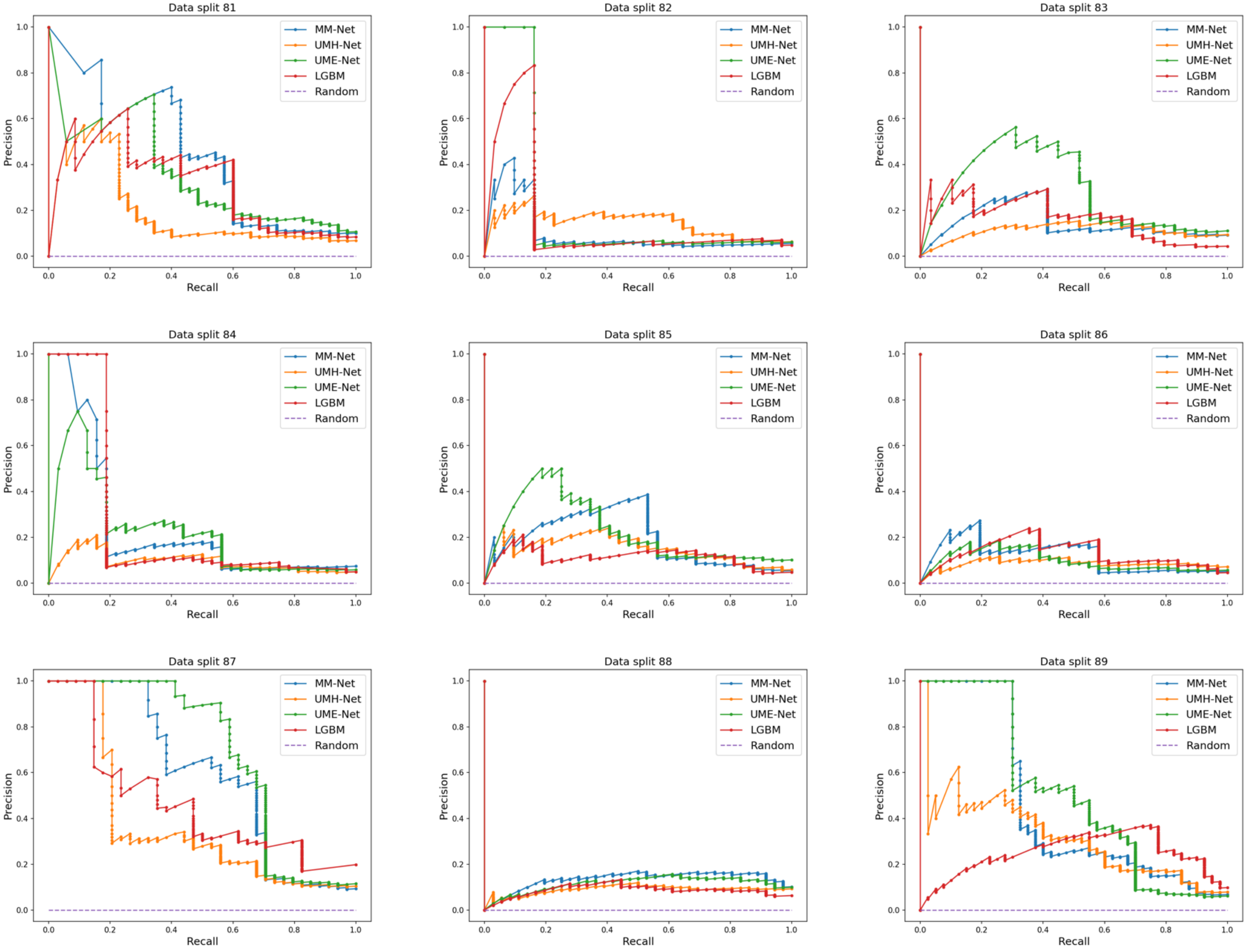
Data split 81
Data split 82
Data split 83
Data split 84
Data split 85
Data split 86
Data split 87
Data split 88
Data split 89
MM-Net
UMH-Net
UME-Net
LGBM
Random
Precision
Recall
0.0
0.2
0.4
0.6
0.8
1.0

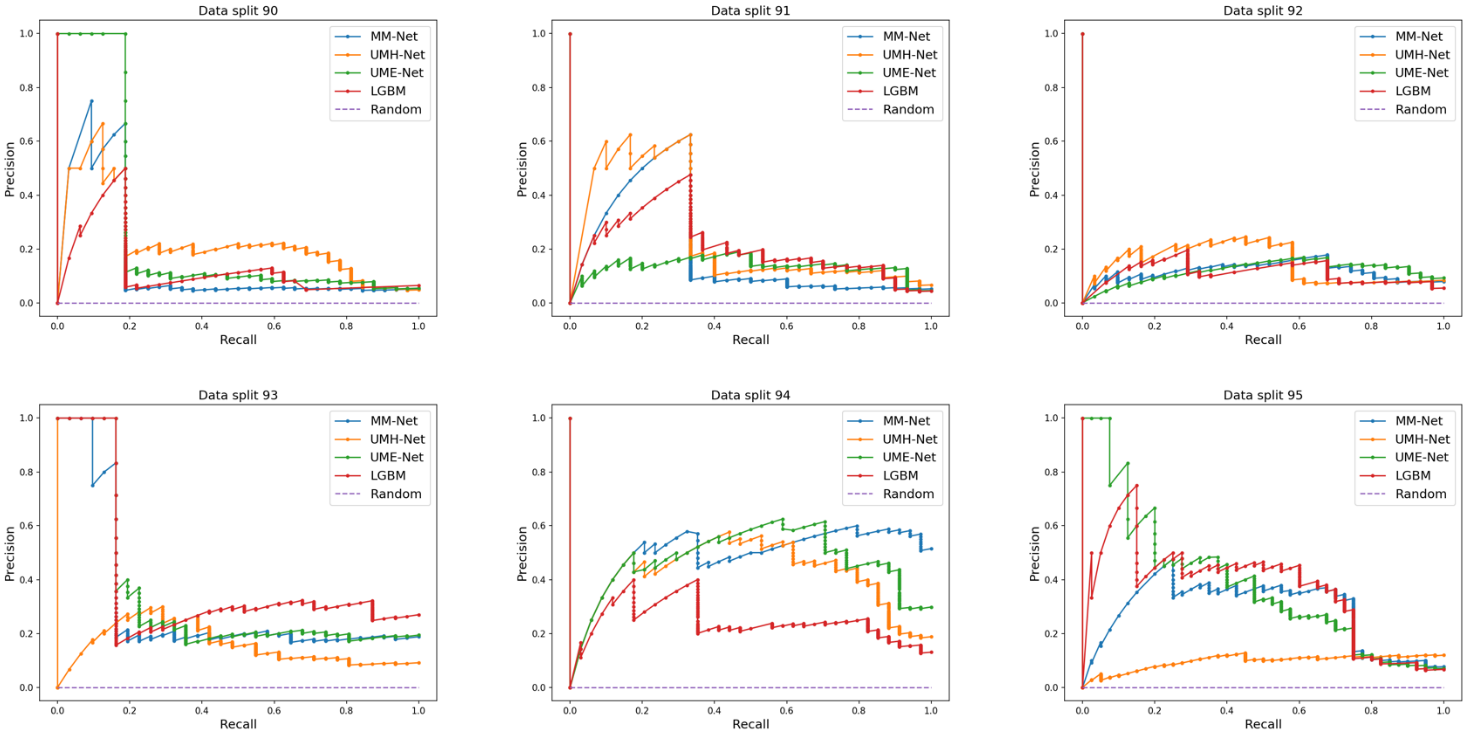

Data split 90
Data split 91
Data split 92
Data split 93
Data split 94
Data split 95
MM-Net
UMH-Net
UME-Net
LGBM
Random
Precision
Recall
0.0
0.2
0.4
0.6
0.8
1.0

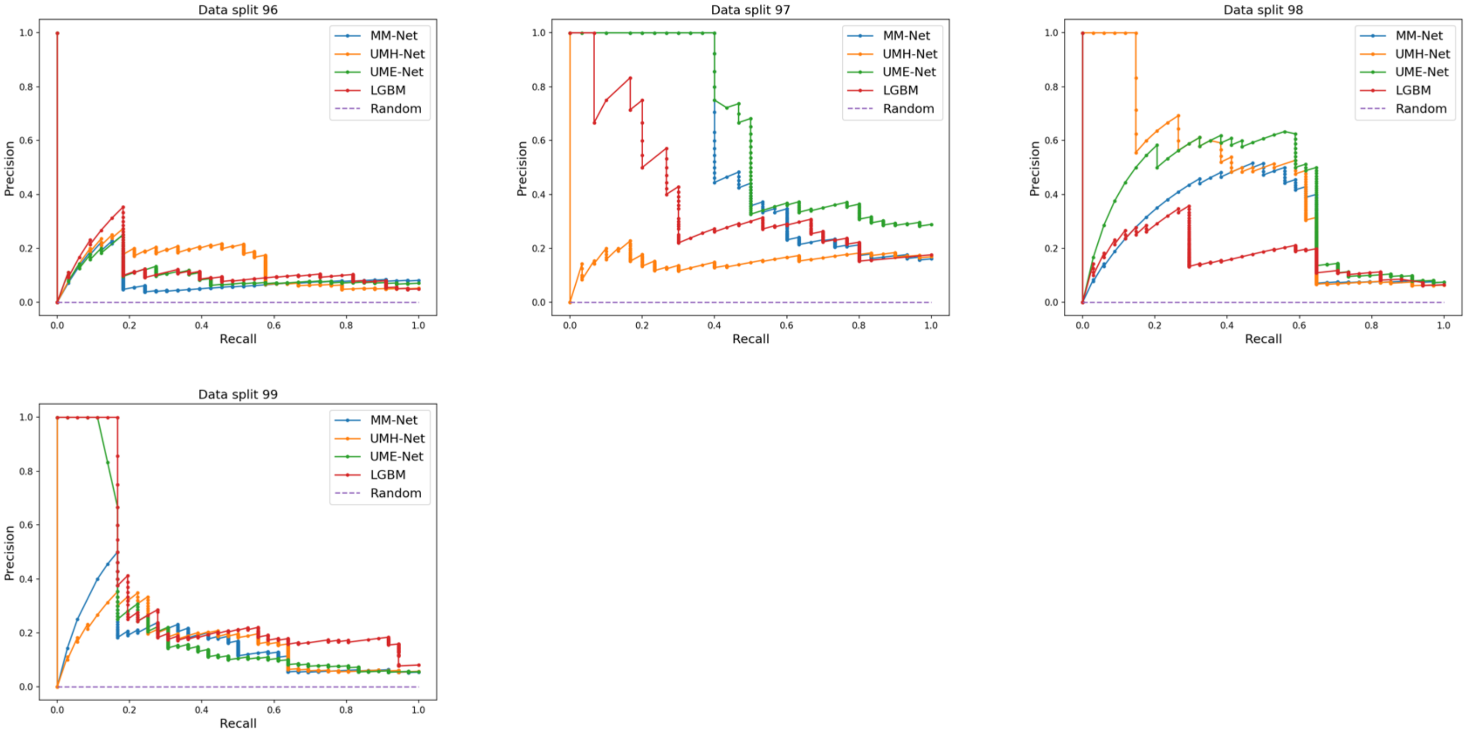
Data split 96
Data split 97
Data split 98
Data split 99
MM-Net
UMH-Net
UME-Net
LGBM
Random
Precision
Recall
0.0
0.2
0.4
0.6
0.8
1.0
